# Ensemble-Level Organization of Human Kinetochores and Evidence for Distinct Tension and Attachment Sensors

**DOI:** 10.1016/j.celrep.2020.107535

**Published:** 2020-04-28

**Authors:** Emanuele Roscioli, Tsvetelina E. Germanova, Christopher A. Smith, Peter A. Embacher, Muriel Erent, Amelia I. Thompson, Nigel J. Burroughs, Andrew D. McAinsh

**Affiliations:** 1 Centre for Mechanochemical Cell Biology, University of Warwick, Coventry, UK; 2 Division of Biomedical Sciences, Warwick Medical School, University of Warwick, Coventry, UK; 3 Mathematics Institute, University of Warwick, Coventry, UK

**Keywords:** mitosis, kinetochore, spindle assembly checkpoint, microtubule dynamics, Ndc80, Knl1, tension, Bayesian inference

## Abstract

Kinetochores are multi-protein machines that form dynamic attachments to microtubules and control chromosome segregation. High fidelity is ensured because kinetochores can monitor attachment status and tension, using this information to activate checkpoints and error-correction mechanisms. To explore how kinetochores achieve this, we used two- and three-color subpixel fluorescence localization to define how proteins from six major complexes (CCAN, MIS12, NDC80, KNL1, RZZ, and SKA) and the checkpoint proteins Bub1, Mad1, and Mad2 are organized in the human kinetochore. This reveals how the outer kinetochore has a high nematic order and is largely invariant to the loss of attachment or tension, except for two mechanical sensors. First, Knl1 unravels to relay tension, and second, NDC80 undergoes jackknifing and loss of nematic order under microtubule detachment, with only the latter wired up to the checkpoint signaling system. This provides insight into how kinetochores integrate mechanical signals to promote error-free chromosome segregation.

## Highlights

• 3D mapping of kinetochore architecture in human RPE1 cells
• Outer kinetochore (NDC80, Mad1, and RZZ) has high nematic order
• NDC80 jackknives and KNL1 unravels upon loss of attachment and tension, respectively
• Recruitment of Mad2 is only coupled to the occupancy sensor (NDC80)

## Graphical Abstract

**Figure:**
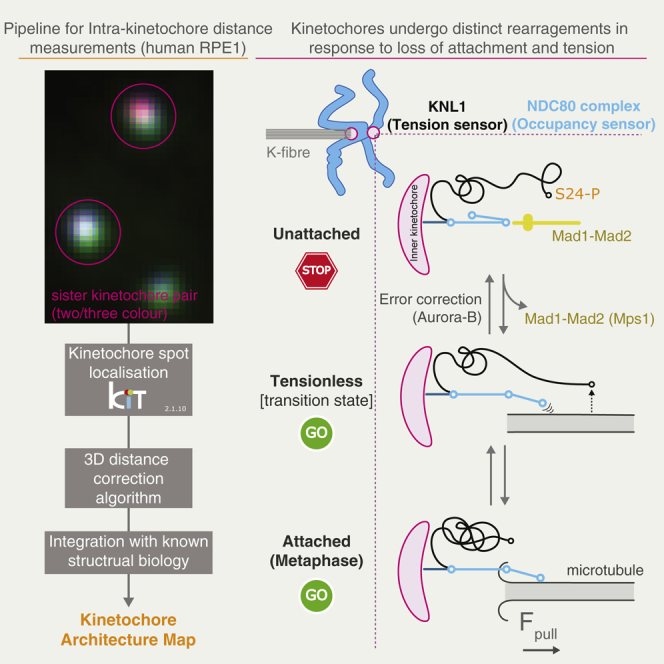

## Teaser

Roscioli et al. use subpixel imaging and computational methods to determine the ensemble-level 3D organization of the human kinetochore. They show how kinetochores undergo distinct rearrangements in response to the loss of attachment and tension.

## Introduction

Human kinetochores are multi-megadalton-sized protein machines that assemble on the centromeres of every sister chromatid and provide an attachment site for the tips of ∼20 dynamic spindle microtubules (the kinetochore [K]-fiber). Kinetochores must coordinate microtubule dynamics within the K-fiber and maintain attachment during phases of growth and shrinkage, thus coupling the energy release from microtubule depolymerization to do work (Auckland and McAinsh, 2015, Rago and Cheeseman, 2013). These kinetochore-microtubule attachments are essential for the accurate segregation of chromosomes in all eukaryotes. However, there is limited understanding of how this machinery adapts to changes in microtubule occupancy and/or the imposition of pushing and pulling forces. These are important questions because a substantial body of work indicates that kinetochores must be able to sense changes in tension and occupancy (Long et al., 2019), sensors that underpin decision making and error correction of the kinetochore.

Classic biophysical experiments established how applying tension to a kinetochore stabilized the attachment to microtubules (Nicklas and Koch, 1969) and that this was coupled to changes in the chemical (phosphorylation) state of the kinetochore (Nicklas et al., 1995). Live cell imaging experiments further show that tension between sister kinetochores (as measured by changes in inter-kinetochore [K-K] distance) can explain the oscillatory movements of bi-oriented kinetochores, being a determinant of directional switching (Burroughs et al., 2015, Wan et al., 2012). Changes in the K-K distance were also implicated in error-correction processes that destabilize improper kinetochore attachments and stabilize bi-orientation (Lampson and Cheeseman, 2011, Tanaka et al., 2005). Recent work has, however, shown that low K-K tension kinetochores do not necessarily trigger error correction (Dudka et al., 2018). Furthermore, the imposition of K-K tension does not appear to be required for silencing of the spindle assembly checkpoint (SAC). By correlating the number of kinetochore-bound microtubules with checkpoint protein recruitment, it appears that the crucial transition to SAC silencing occurs at approximately half-maximal occupancy (Dudka et al., 2018, Etemad et al., 2015, Etemad et al., 2019, Kuhn and Dumont, 2017). Kinetochores thus appear to be able to “count” the number of bound microtubules.

Intra-kinetochore tension may generate key mechanical signals that are sensitive to changes in microtubule attachment and/or the imposition of force (for review, see Maresca and Salmon, 2010). Intra-kinetochore tension refers to the measurement of changes in distance between two components of the kinetochore labeled with different fluorophores (denoted by Δ). Initial pioneering experiments showed that increased Δ and not the K-K tension correlates with SAC silencing (Maresca and Salmon, 2009, Uchida et al., 2009, Wan et al., 2009). However, there is also evidence that high Δ is not always necessary for SAC silencing (Etemad et al., 2015, Magidson et al., 2016, Tauchman et al., 2015). It thus remains uncertain how the kinetochore monitors change in occupancy and whether it can sense changes in tension at all. One idea is that there are kinetochore conformations or tensile elements that would function as tension and/or attachment sensors.

While X-ray crystallography and electron microscopy are beginning to provide a structural view of the kinetochore (Hamilton et al., 2019, Pesenti et al., 2016, Welburn and Cheeseman, 2008), this approach is limited to subsets of kinetochore components and does not allow the impact of microtubule binding and forces to be easily determined. Furthermore, these approaches are limited to single assemblies, while the human kinetochore in a living cell incorporates multiple microtubule attachment sites (∼20) populated with multiple copies of each kinetochore complex (Huis In ’t Veld et al., 2016, Johnston et al., 2010, Suzuki et al., 2011). Thus, the *in vivo* higher-order, ensemble-level organization of the human kinetochore remains out of reach.

## Results

### Measurement of 3D Euclidian Distances between Kinetochore Proteins

**Figure 1:**
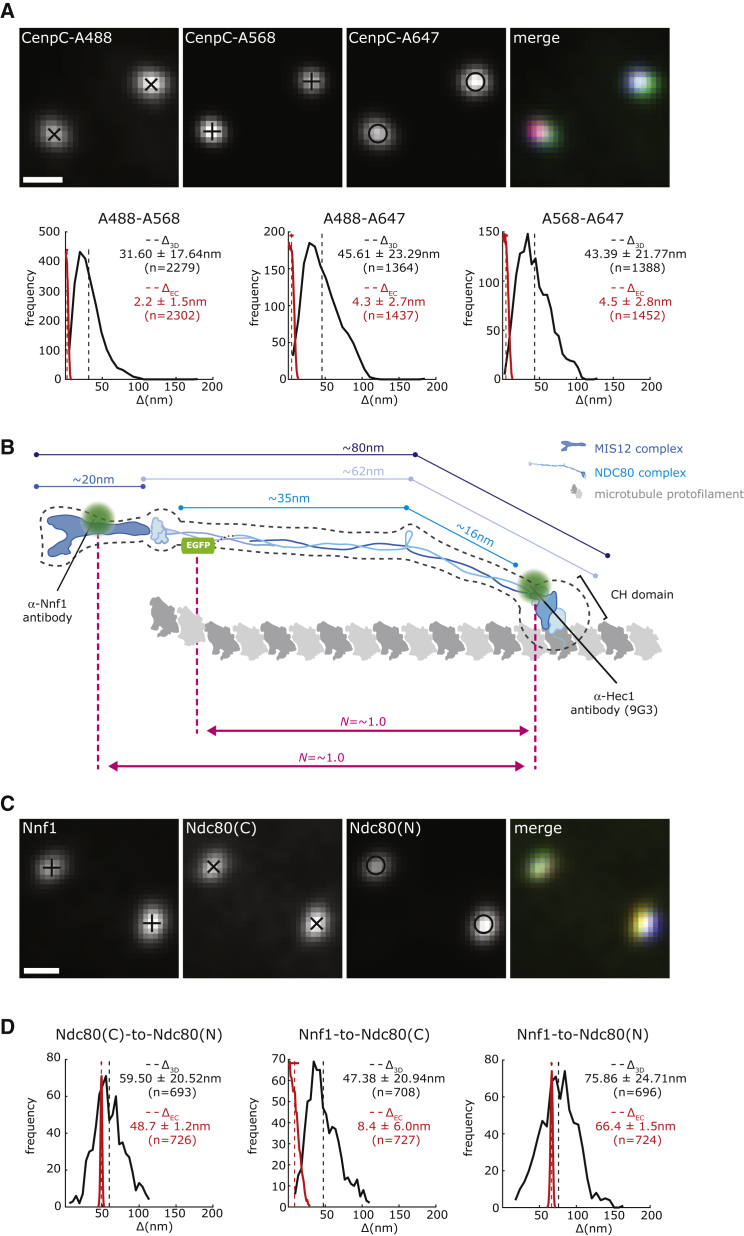
Δ_3D_ Measurements Overestimate the True Δ Distance (A) Kinetochores stained with anti-CenpC primary antibody and a mixture of Alexa488 (A488), Alexa568 (A568), and Alexa647 (A647)-conjugated secondary antibodies. Histograms of Δ_3D_ (black) and inferred Δ_EC_ (red) distances between the indicated fluorophores, mean (dashed line), and SD (horizontal bar for Δ_EC_) values are indicated at right. (B) Schematic representation of MIS12 complex (dark blue) and NDC80 complex (light blue) bound to a microtubule protofilament (dark gray). Assembly organization and size are based on electron microscopy (black dotted line) and crystallography studies (see Methods S1 for details). Approximate antibody binding sites (or EGFP tagging) are indicated with green dots. Distances used for nematic order (*N*) calculation are indicated in pink (see Table S2). (C) Kinetochores stained with anti-Nnf1 and anti-Hec1(9G3) antibodies and RPE1 Ndc80-EGFP cells. (D) Histograms of the Ndc80(C) to Ndc80(N), Nnf1 to Ndc80(C), and Nnf1 to Ndc80(N) Δ_3D_ and the Δ_EC_ distances measured in Ndc80-EGFP cells; means and SDs are displayed as in (A). Scale bars, 500 nm.

To obtain insight into the 3-dimensional (3D) nanoscale architecture of the human kinetochore, we deployed an image acquisition and computational pipeline that outputs the 3D Euclidian distances (Δ_3D_) between differentially labeled kinetochore proteins in near-diploid hTERT-RPE1 cells (referred to hereafter as RPE1; Smith et al., 2016). One limitation of this approach is the overestimation of mean distances (Suzuki et al., 2018). This is because Euclidian distances cannot be negative leading to a positive bias in the Δ_3D_ distribution; in other words, distances are overestimated (Figure 1A; Table S1; Methods S1). To correct for this bias, we developed an algorithm to infer the true Euclidian distance (Δ_EC_) between two fluorophores. This algorithm goes beyond previous methods (Churchman et al., 2005, Suzuki et al., 2018) by taking into account the anisotropic point spread function in 3D datasets (see Methods S1 for details). To test the accuracy of this method, we fixed cells and stained them with an anti-CenpC antibody that recognizes the amino-terminal region of the protein (amino acids [aa] 1–426), binding to a site preceding the region binding CenpA nucleosomes. This primary antibody was then detected using a mixture of 3 different secondary antibodies (conjugated to Alexa Fluor 488, 568, and 647; Figure 1A). The Euclidean bias-corrected delta distances were 2.2 ± 1.5 nm (mean ± standard deviation; n = 2,302; Alexa Fluor 488–568), 4.3 ± 2.7 nm (n = 1,437; Alexa Fluor 488–647), and 4.5 ± 2.8 nm (n = 1,452; Alexa Fluor 568–647), considerably smaller than the corresponding Δ_3D_ (Figure 1A; Table S1).

These data confirm that we can measure the 3D nanoscale distances between 3 different fluorophores within the human kinetochore with an accuracy on the scale of 2–4 nm. As a second test, the amino-terminal end of the Ndc80 subunit (of the NDC80 complex) was marked with the 9G3 monoclonal antibody that recognizes the N-terminal globular domain of Hec1 (aa 200–215; DeLuca et al., 2006; hereafter referred to as Ndc80(N)) and the carboxy-terminal end was marked with EGFP (Ndc80(C); Figures 1B and 1C). To label the Ndc80 C-terminal end, we inserted EGFP at the endogenous locus by CRISPR-Cas9 gene editing in RPE1 cells (cell line MC191; see Methods S1 and Figure S2 for details). The Δ_EC_ distance from Ndc80(C) to Ndc80(N) was 48.7 ± 1.2 nm (n = 726; Figure 1D; Table S1). Because there is substantial structural information describing the NDC80 complex *in vitro*, it has been used as a “molecular ruler” to validate 2 color fluorescence localization methods (Suzuki et al., 2018). Our Δ_EC_ measurements are close to the 51 nm distance estimated using negative stain electron microscopy (EM) (Huis In ’t Veld et al., 2016, Wei et al., 2005; Figure 1B). As expected, the Δ_3D_ measurement is an overestimate (59.50 ± 20.52 nm, n = 693; Figure 1D; Table S1), while the Δ_1D_ is typically an underestimate (33.37 ± 14.48 nm, n = 283; Figure S1A; Table S1; Smith et al., 2016). We next measured the Δ_EC_ distance between the MIS12 complex subunit Nnf1 and Ndc80(N) (Figures 1B and 1C) and obtained a value of 66.4 ± 1.5 nm (n = 724; Figure 1D; Table S1). This is similar to the distance measured in parental cells that did not have EGFP knocked in to the *NDC80* locus (Δ_EC_, 61.5 ± 0.8 nm, n = 1,748; Figure S1D; Table S1). Again, these distances are very close to the predicted distance (∼65 nm) that can be estimated from EM studies (Petrovic et al., 2010, Screpanti et al., 2011). Finally, as expected from *in vitro* reconstitution experiments (Helgeson et al., 2018, Huis In ’t Veld et al., 2019) the SKA complex (Ska3) was located proximal to the Ndc80 amino terminus (Table S1). Overall, these data confirm the accuracy of our method and the importance of correcting intra-kinetochore distance measurements.

### The Inner Kinetochore Is Offset from the Outer Kinetochore

**Figure 2:**
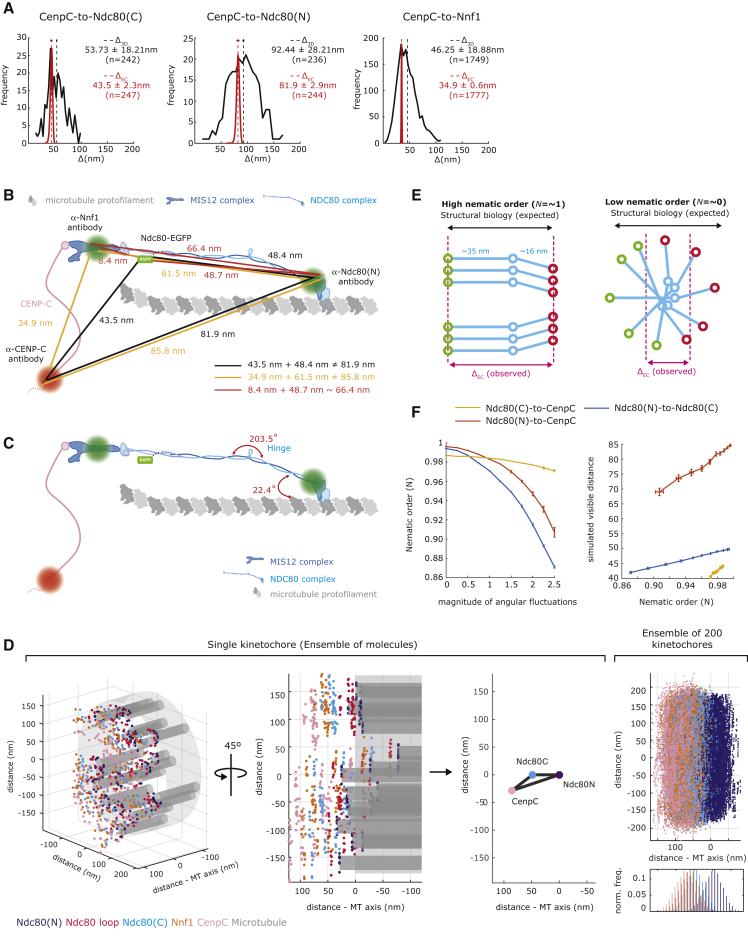
Simulations of the Architecture of a Bi-oriented Kinetochore (A) Histograms of Δ_3D_ and Δ_EC_ distances for CenpC to Ndc80(C) and CenpC to Ndc80(N) (Ndc80-EGFP cells) and for CenpC to Nnf1, mean (dashed line), and SD (horizontal bar) values are indicated at right. (B) Schematic showing the inferred architecture of CenpC-MIS12-NDC80. Approximate antibody binding sites (or EGFP tagging) are indicated with green dots. Lines indicate Δ_EC_ mean values (dotted lines show SDs) obtained in 3-fluorophore experiments: CenpC-Ndc80(C)-Ndc80(N) (black), CenpC-Nnf1-Ndc80(N) (orange), and Nnf1-Ndc80(C)-Ndc80(N) (red). (C) Schematic of CenpC-MIS12-NDC80 showing best-fitted angles (red) from simulations; see Methods S1. (D) Simulated kinetochore organization. Left and center left: 2 perspectives of the same simulated kinetochore; each dot represents a single protein at the specified position (labeled by color). Center right: averages of the simulated position of each marker shown in the plane of the CenpC-Ndc80(N)-Ndc80(C) triangle. Right: an ensemble of 200 simulated kinetochores aligned along their microtubule axis and to the mean of their Ndc80(N) markers. Histogram of the marker density along K-fiber for the above plot. (E) Schematic representation of NDC80 complexes under high (N = ~1) and low (N = ~0) nematic order. Circles represent Ndc80(C) (green), Ndc80 loop (blue), and Ndc80(N) (red). (F) Left: change in the nematic order of the NDC80 complex population with respect to the magnitude of Ndc80 loop stochastic angular fluctuations. Right: average distance between the indicated molecular markers against the nematic order. Simulations based on 200 kinetochores.

Concurrent with measuring the distance between Ndc80(C), Ndc80(N), and Nnf1, we also determined an inner kinetochore position using the CenpC antibody used in Figure 1A. We measured the distances from the amino- and carboxy-ends of Ndc80 to CenpC in a 3-fluorophores experiment and obtained a triangle with side lengths of 43.5 ± 2.3 nm (n = 247), 81.9 ± 2.9 nm (n = 244), and 48.4 ± 3.1 nm (n = 247; Figures 2A, 2B, and S1B; Table S1). These 3 distances are not compatible with collinearity (p = 0.019, z = 2.07; Figure 2B; Table S1). To further substantiate a lack of collinearity, we also measured the distances from Ndc80(N) and CenpC to Nnf1 in parental cells (Figure S1C). These measurements also indicate a lack of collinearity (p = 6 × 10^−17^, z = 8.28) with side lengths of 34.9 ± 0.6 nm (n = 1,777), 85.8 ± 0.8 nm (n = 1,754), and 61.5 ± 0.8 nm (n = 1,748; Figures 2A, 2B, and S1D; Table S1). This contrasts with distances between Nnf1-Ndc80(C)-Ndc80(N), which are collinear to our accuracy (p = 0.07, z = 1.47, distances 48.7 ± 1.2 nm [n = 726], 8.4 ± 6.0 nm [n = 727], and 66.4 ± 1.5 nm [n = 724]; Figures 1D, 2B, and S1A; Table S1). The CenpC position (inner kinetochore) must therefore be offset (on average) from the axis defined by the Nnf1-Ndc80(C)-Ndc80(N) axis.

### Visualization of Kinetochore Ensemble Organization

Each kinetochore contains multiple copies of each protein per microtubule and multiple microtubule binding sites. Our experiments thus measure the ensemble average position of the tagged proteins and not the distance between single molecules. To understand the observed lack of collinearity and interpret our distances in the context of known structural information, we built a computational simulation. Our simulations incorporate (1) known structural biology on NDC80-MIS12, (2) information from EM data of K-fiber and kinetochore organization, and (3) measurements of kinetochore protein numbers per microtubule. We optimized unknown parameters by fitting the simulations to our measured triangle CenpC-Ndc80(C)-Ndc80(N). Crucially, we fit the NDC80 complex hinge angle and the elevation of the NDC80 complex short arm (Ndc80(N)-to-Ndc80(hinge)) with the microtubule. Best fits are 203.5° and 22.4°, respectively (Figure 2C; see Methods S1); hinge angles >180° have been observed (Scarborough et al., 2019, Wang et al., 2008), while the latter is compatible with the range determined from EM and crystal structure studies (Ciferri et al., 2008, Wilson-Kubalek et al., 2008). Figure 2D (left and center left panels) shows a simulation of a single kinetochore bound to a K-fiber (gray) with the simulated positions of CenpC (pink), Ndc80(C) (light blue), Ndc80 loop (red), Ndc80(N) (blue), and Nnf1 (orange) at single molecules. The center right panel displays the average of the simulated position of three markers showing the offset of CenpC with respect to the Ndc80 complex. Averaging across multiple simulated kinetochores reveals a multi-layered structure with tight localization and sequential ordering of kinetochore components along the K-fiber axis, and an extensive spread transverse to the axis that is suggestive of discs (Figure 2D, right panel). Our simulations indicate that moderate levels of flexibility of the Ndc80 microtubule attachment angle and orientation can produce a lack of collinearity between the average positions of CenpC, Nnf1/Ndc80(C), and Ndc80(N). In addition, the broad transverse dimension allows even small re-distributions of the CenpC population to give rise to an off-axis location. Thus, changes in the distribution of the CenpC marker, potentially due to CenpC’s inherent flexibility, are sufficient to explain the lack of collinearity measured *in vivo*.

The simulations also revealed ordered alignment of kinetochore molecules along an internal axis. We sought to quantify the degree of alignment of individual NDC80 complexes. Because these complexes are rod-like with head-tail asymmetry, we can calculate the nematic order (N), which is a measure of the mean degree of filament alignment along a director (de Gennes and Prost, 1993, Doostmohammadi et al., 2018) and related to our distance measurements by N = Δ_EC_/Δ_structural_ (see Methods S1). Here, N = ∼0 indicates no alignment (low nematic order), and N = ∼1 is perfect parallel alignment of vectors (high nematic order; Figure 2E). At high alignment of the NDC80 complexes within the ensemble (i.e., high nematic order), the structural data and our *in vivo* distance measurements should match, while as nematic order decreases, the measured distance of the ensemble decreases (Figure 2E). We find that for both the Ndc80(C) to Ndc80(N) and Nnf1 to Ndc80(N) linkages, N = ∼1 (Figure 1B; Table S2). This indicates that in the microtubule-attached state, the MIS12-NDC80 complex assemblies are highly ordered and that Δ_EC_ distances reflect the underlying molecular organization as implicitly assumed in using the NDC80 complex as a molecular ruler (Suzuki et al., 2018). We also investigated how increasing the fluctuations in the hinge angle and Ndc80 orientation decreased the nematic order (Figure 2F, left panel) and correspondingly decreased the average Δ_EC_ distance (Figure 2F, right panel). This further shows how the angular degrees of freedom, such as the NDC80 hinge (Methods S1) are in effect highly constrained to produce high alignment of the NDC80 complexes. Thus, our simulations and data indicate that at this resolution, the kinetochore is organized along an internal axis with high nematic order in the outer plate (Table S2).

### Linking the Inner and Outer Kinetochores: CenpT and CenpC-Mis12 Linkages Both Position NDC80 in the Same Spatial Domain

**Figure 3:**
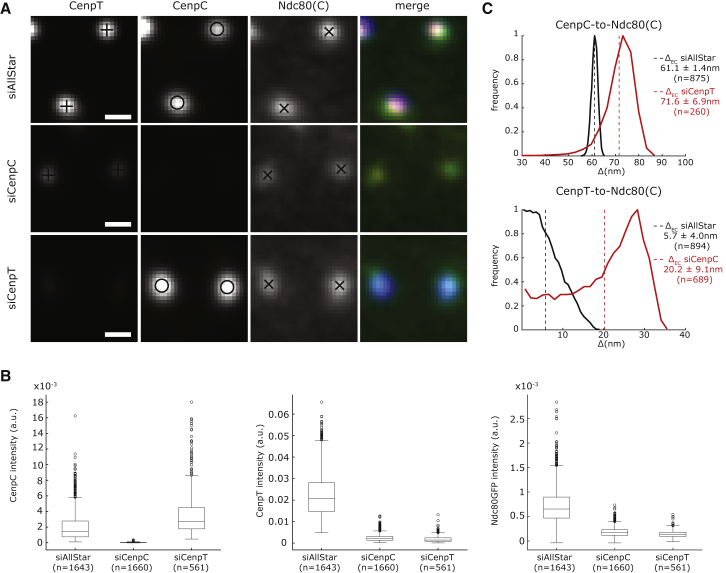
The CenpC and CenpT Linkers Contribute to a Single Spatial Population of NDC80 Complexes (A) Kinetochores stained with anti-CenpT and anti-CenpC antibodies in Ndc80-EGFP cells transfected with small interfering AllStar (siAllStar) (control) and siRNA against CenpC and CenpT. Scale bar, 500 nm. (B) Box and whiskers plots of CenpC, CenpT, and Ndc80-EGFP kinetochore intensity in the different RNAi conditions; signals are background subtracted. (C) Histograms of CenpC to Ndc80(C) and CenpT to Ndc80(C) Δ_EC_ distances measured in Ndc80-EGFP cells treated as in (A); mean (dashed line) and SD values are indicated at right.

It is well established that NDC80 complexes are not only targeted to kinetochores through CenpC-Mis12 linkages but also through CenpT (Gascoigne et al., 2011, Klare et al., 2015); each linker is responsible for recruiting ∼50% of the Ndc80 molecules (Johnston et al., 2010). We determined the CenpT position using an antibody, which recognizes the non-histone fold region that extends and interacts with the NDC80 complex. This CenpT epitope was positioned close (5.7 ± 4.0 nm, n = 894) to the Ndc80 C terminus and 47.9 ± 1.6 nm (n = 882) from CenpC (Figure 3C; Table S1). This suggested that the NDC80 complexes tethered through CenpT or CenpC-MIS12 are not spatially separated within the kinetochore. To further investigate, we depleted each linker by RNAi. Depletion of CenpT resulted in the loss of >93% of the CenpT molecules from kinetochores and an increase in the number of CenpC molecules (Figures 3A and 3B). However, the distance between the remaining endogenous Ndc80-EGFP (∼20%) and CenpC molecules increased by only 10.5 ± 7.0 nm compared to control cells (Figure 3C; Table S1). The reciprocal experiments (CenpC depleted by >96%) resulted in the unbinding of CenpT (by ∼90%) and Ndc80-EGFP (by ∼75%; Figures 3A and 3B). The distance between the residual CenpT and Ndc80(C) also increased by 14.5 ± 9.9 nm (Figure 3C; Table S1). These data support the idea of a single NDC80 complex population and confirm that the linkers are required for normal kinetochore integrity and composition (Klare et al., 2015, Suzuki et al., 2014).

### Jackknifing of NDC80 Complexes Follows Treatment with Nocodazole, but Not Taxol

We next investigated how the structural organization and geometry of the kinetochore respond to a loss of attachment or tension. To do this, we treated the RPE1 cell lines with either (1) 3.3 μM nocodazole for 2 h and confirmed by tubulin staining that all of the kinetochore-microtubule populations were eliminated and that the 3D K-K distance (CenpC to CenpC) was reduced (0.93 ± 0.005 μm, n = 2,537, means ± SEMs), or (2) 1 μM taxol for 15 min, which reduces the K-K distance to nearly rest length (0.96 ± 0.004 μm, n = 1,931, means ± SEMs), but leaves the majority of kinetochores associated with microtubules and aligned on the metaphase plate (Figure S3A). By measuring microtubule dynamics using photoactivatable PA-EGFP-alpha-tubulin, we also confirmed that 1 μM taxol largely eliminated microtubule dynamics (Amaro et al., 2010), consistent with the loss of tension (Figure S3).

**Figure 4:**
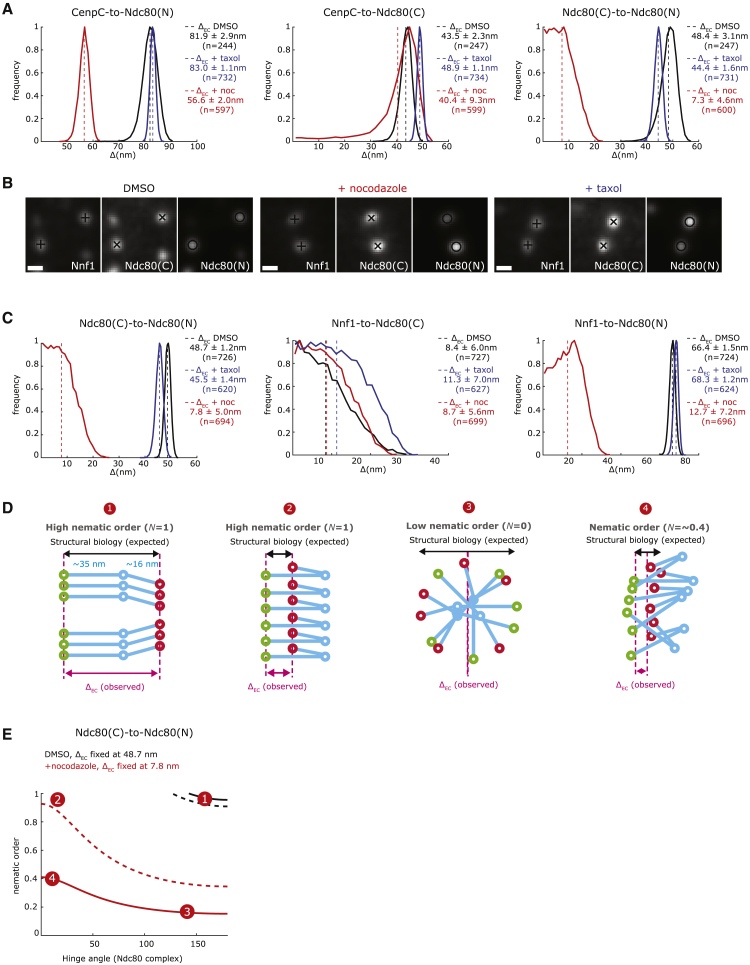
Loss of Microtubule Attachment and Stabilization of Microtubule Dynamics Induce Different Organization of Ndc80 (A) Histograms of CenpC to Ndc80(N), CenpC to Ndc80(C), and CenpC to Ndc80(N) Δ_EC_ distances in Ndc80-EGFP cells treated with 3.3 μM nocodazole for 2 h, 1 μM taxol for 15 min, or DMSO; mean (dashed line) and SD values are indicated at right. (B) Kinetochores stained with anti-Nnf1 and anti-Hec1(9G3) antibodies and in Ndc80-EGFP cells treated as in (A). Scale bar, 500 nm. (C) Histograms of Ndc80(C) to Ndc80(N), Nnf1 to Ndc80(C), and Nnf1-to-Ndc80(N) Δ_EC_ distance in Ndc80-EGFP cells treated as in (A); mean (dashed line) and SD values are indicated at right. (D) Four models of different structural and nematic organizations of NDC80. Circles represent Ndc80(C) (green), Ndc80 loop (blue), and Ndc80(N) (red). For each model, the nematic order (gray), the distance expected from structural information (black), and the expected Δ_EC_ (pink) are indicated. (E) Data-constrained nematic order and hinge angle of the NDC80 complex. For each hinge angle, the nematic order is calculated that is consistent with the observed Δ_EC_, 48.7 nm (DMSO) and 7.8 nm (nocodazole) for the Ndc80(C) to Ndc80(N) linkage. Dashed lines show 95% confidence intervals. Numbered circles annotate the approximate position of each model on the graph.

We measured the geometry of the CenpC-MIS12-NDC80 assembly; the distances from CenpC to Ndc80(C) and Nnf1 were largely unchanged in nocodazole and taxol (Figures 4A, S4A, and S4B; Table S1). This is consistent with previous work reporting that the inner kinetochore does not collapse following microtubule detachment (Smith et al., 2016, Suzuki et al., 2018). However, the CenpC to Ndc80(N) distance was reduced by 25.3 ± 3.5 nm following treatment with nocodazole (Ndc80-EGFP cells; Figure 4A; similar to the reduction of 30.1 ± 3.3 nm observed in parental cells; Figure S4B; Table S1). Consistently, the Ndc80(C) to Ndc80(N) distance decreased by 41.1 ± 5.5 nm (85%) in unattached kinetochores (Figure 4A). We also measured the distances between Nnf1, Ndc80(C), and Ndc80(N) (Figure 4B). This triangulation confirms that there is a substantial reduction of 40.9 ± 5.1 nm (84%) in the Ndc80(C) to Ndc80(N) linkage under nocodazole treatment (Figure 4C; Table S1). In taxol, the Δ_EC_ between Ndc80(C) and Ndc80(N) remained the same as in the control (Figures 4A and 4C; Table S1). This suggests that the Ndc80 N terminus does not move inward following taxol treatment and the NDC80 complex remains in its straight configuration with a high nematic order, N = ∼1 (Table S2). These data provide evidence that the kinetochore responds differentially to the loss of attachment and tension.

Next, we sought to investigate the underlying cause of the ∼85% reduction in the distance between Ndc80 C and N termini observed under nocodazole treatment. Previous studies have suggested that the Ndc80 loop acts as a pivoting point and thus we asked whether the distance reduction can be explained by conformational change in the Ndc80 complex such that the Ndc80 N terminus is brought into close proximity to the C terminus (we call this jackknifing; Figure 4D, model 2). Ndc80 structural data suggest that the expected distance between Ndc80 C and N termini upon full closure of the N terminus is ∼19 nm, and thus is insufficient to explain the measured 7.8 nm distance in nocodazole. Since the measurements reflect kinetochore ensemble organization, the change could alternatively be explained by the disorganization of the Ndc80 molecules (i.e., loss of nematic order) or a combination of Ndc80 jackknifing and disorganization. Our analysis shows that in the absence of Ndc80 jackknifing (i.e., if Ndc80 maintains its straight conformation as in DMSO), a nematic order decrease from ∼1 observed in DMSO (Figures 4D and 4E, model 1) to <0.2 is required to explain the observed 7.8 nm distance between Ndc80 C and N termini (Figures 4D and 4E, model 3). Alternatively, if Ndc80 undergoes full jackknifing then a loss of order to 0.4 is still required to explain the measured 7.8 nm distance (Figures 4D and 4E, model 4). We favor this model (see Discussion for details) and hereafter refer to these changes in NDC80 organization as jackknifing.

### NDC80 Jackknifing Marks Spindle Checkpoint Active Kinetochores

**Figure 5:**
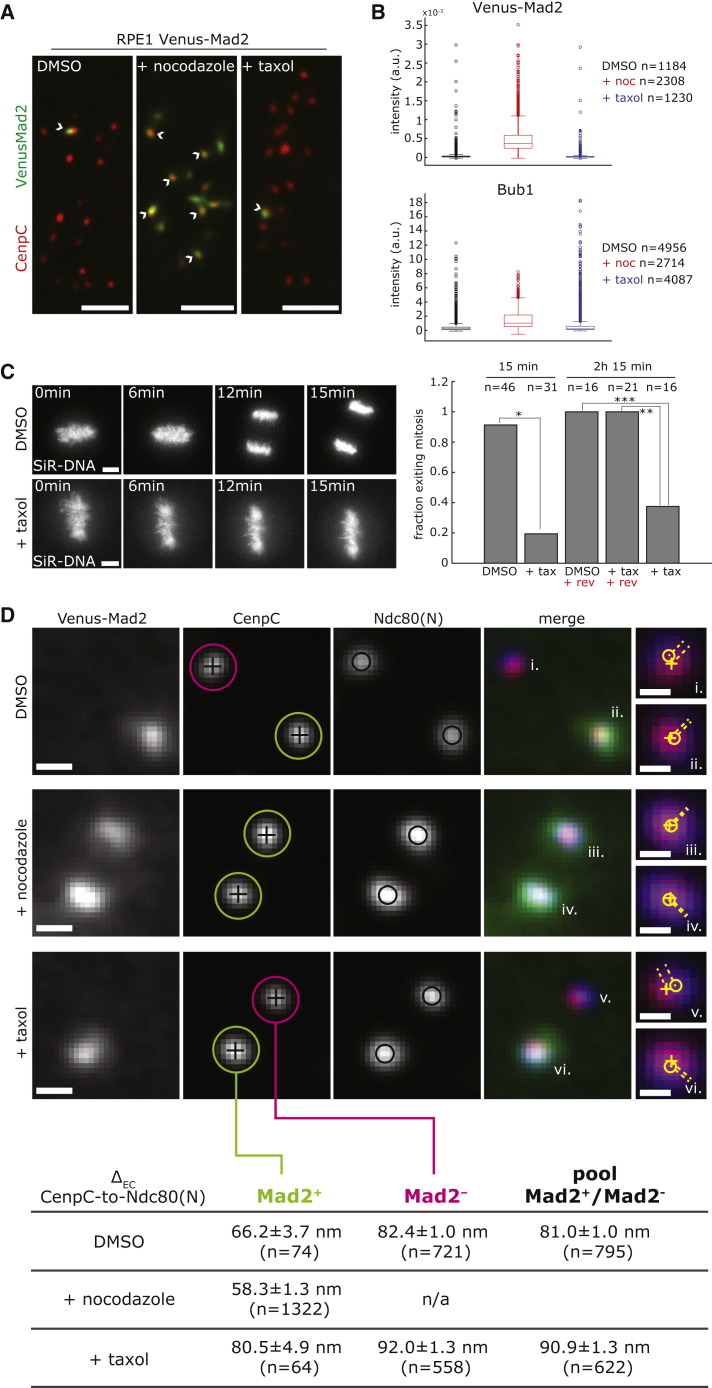
Activation of the Spindle Assembly Checkpoint Correlates with NDC80 Conformational Change (A) Images of Venus-Mad2 cells stained with anti-CenpC antibody and treated with 3.3 μM nocodazole for 2 h, 1 μM taxol for 15 min, or DMSO. White arrows indicate Mad2^+^ kinetochores. Scale bar, 3 μm. (B) Box and whisker plots of Venus-Mad2 and Bub1 kinetochore intensity in cells treated as in (A). All of the intensities are background subtracted. The Bub1 signal is normalized to the CenpC signal. (C) Images of cells treated with 1 μM taxol and DMSO for 15 min and stained with SiR-DNA. Scale bars, 5 μm. Bar chart indicates the fraction of cells exiting mitosis within the indicated imaging time, after DMSO, taxol (+tax), and 1 μM reversine (+rev) treatment (Figure S5B; Method Details). Fisher’s exact test indicates that the differences are significant with a 99% confidence interval: ^∗^p = 7.7 × 10^−12^; ^∗∗^p = 2.3 × 10^−5^; ^∗∗∗^p = 1.2 × 10^−4^. (D) Example images of kinetochores stained with anti-CenpC and anti-Hec1(9G3) antibodies in RPE1 Venus-Mad2 cells treated as in (A). Scale bar, 500 nm. Table S1 displays the Δ_EC_ (mean ± SD) between CenpC and Ndc80(N) in Venus-Mad2^+^ (green circle), Venus-Mad2^−^ (pink circles) kinetochores, and pool samples in the 3 conditions. Insets (i–vi) show the distances (yellow dotted lines) between CenpC (+) and Ndc80 (○) in the indicated kinetochores on the XY plane. Scale bar in insets, 250 nm.

Does jackknifing within the outer kinetochore plate relate to checkpoint signaling from the kinetochore? For these experiments, we used cells in which Mad2 was labeled at its endogenous locus with Venus (Collin et al., 2013). As expected, nocodazole treatment (unattached kinetochores) led to an increase in Venus-Mad2 and Bub1 kinetochore binding on most kinetochores, while after taxol treatment, the average levels of these proteins were similar (Figures 5A, 5B, and S5A). However, in taxol and DMSO, there were multiple outliers (Figure 5B), suggesting the presence of a Mad2-positive subpopulation, as previously reported (Magidson et al., 2016). In addition, we found that treatment with 1 μM taxol prevents cells in metaphase from exiting mitosis unless 1 μM reversine (Mps1 inhibitor) is added to the media to override the spindle checkpoint (Figures 5C and S5B). These experiments indicate that the spindle checkpoint is active following treatment with 1 μM taxol and suggest that the few Mad2-signaling kinetochores may be responsible. To understand how the Ndc80 jackknifing relates to Mad2 recruitment, we separated our kinetochore spots into a Venus-Mad2-positive (Mad2^+^) and -negative (Mad2^−^) population, and then determined the distance from CenpC to Ndc80(N) (Figure 5D). The CenpC to Ndc80(N) distances were significantly different in these 2 populations (p = 2.4 × 10^−5^), with the distance in the Mad2^+^ population (66.2 ± 3.7 nm) nearly identical to that of nocodazole-treated cells (which were all Mad2^+^, Δ_EC_ = 58.3 ± 1.3 nm, p = 0.044). Furthermore, the 16.2 ± 3.8 nm difference in the CenpC to Ndc80(N) between Mad2^−^ and Mad2^+^ in DMSO (Figure 5D; Table S1) is consistent with the jackknifing of Ndc80 under the lack of attachment in Mad2^+^ (compare to the Δ_EC_ decrease of 25.3 ± 3.5 nm under nocodazole, p = 0.039), suggesting that there is a high correlation between Mad2^+^/Mad2^−^ and Ndc80 being in the jackknife or straight conformations (nocodazole versus DMSO, respectively). Under taxol, we could also detect an inward movement of 11.5 ± 5.1 nm of the Ndc80(N) in the Mad2^+^ versus Mad2^−^ subpopulations (Figure 5D). These correlations are likely imperfect because the Mad2^+^/Mad2^−^ populations are also heterogeneous with respect to attachment status and include partial jackknifing states as error correction proceeds.

These data indicate that we can detect the inward movement of Ndc80(N) at Mad2^+^ kinetochores in control metaphase cells, not just under nocodazole treatment. These data clearly demonstrate that checkpoint activation (in terms of Mad2 recruitment) occurs at kinetochores that are in the unattached conformation (Ndc80 jackknifed). Moreover, our data show how most kinetochores in 1 μM taxol have a low K-K distance without triggering the recruitment of Mad2. We next reasoned that if the Ndc80 jackknifing is downstream of checkpoint activation, then it should not occur when Mps1 kinase activity is inhibited. The treatment of cells with 1 μM reversine eliminated the Mad1-pT716 substrate signal (Allan et al., 2020), but had no effect on Ndc80(N) movement in the presence of nocodazole (and MG132 to prevent entry to anaphase; Figure S5C), suggesting that jackknifing is independent from Mps1 activity.

### 3D Mapping of the Key Checkpoint Protein Platforms Bub1 and RZZ

**Figure 6:**
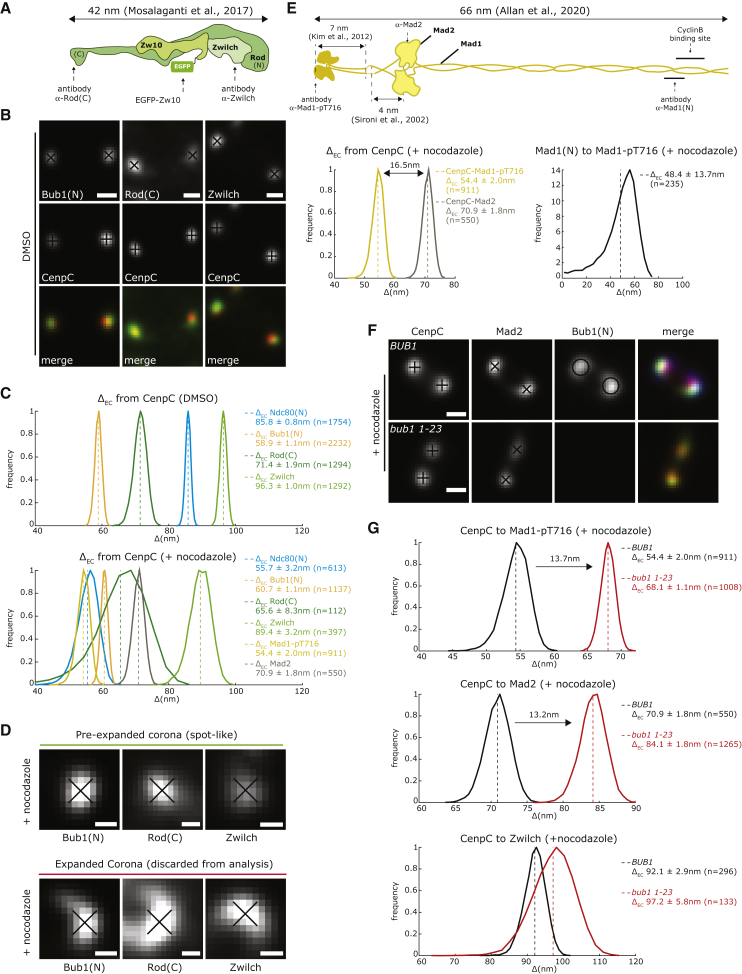
Mad1:Mad2 Binds Different Kinetochore Sites upon Activation of the Spindle Assembly Checkpoint (A) Scale model of RZZ and approximate positions of anti-Rod(C) and anti-Zwilch antibodies and EGFP-Zw10. (B) Kinetochore pairs stained with anti-CenpC antibody in combination with anti-Bub1, anti-Rod, or anti-Zwilch antibodies in DMSO-treated cells. Scale bar, 500 nm. (C) Histograms of Δ_EC_ between CenpC and Ndc80(N), Bub1, Rod, and Zwilch in DMSO- and nocodazole-treated cells. Mad1-pT716 and Mad2 positions are shown for nocodazole only. Mean (dashed line) and SD values are indicated at right. (D) Examples of spot-like and expanded kinetochores. Kinetochores are stained with anti-Bub1, anti-Rod, and anti-Zwilch antibodies in cells treated with 3.3 μM nocodazole. Scale bars, 250 nm. (E) Top: schematic indicating the Mad1:Mad2 complex size and the approximate binding positions of anti-Mad1-pT716; anti-Mad1 directed against aa 77–115, referred to as Mad1(N); and anti-Mad2 antibodies used in this study. Bottom: histograms of the Δ_EC_ values between CenpC to Mad1-pT716 and CenpC to Mad2 (left), and Mad1(N) to- Mad1-pT716 (right). Mean (dashed line) and SD values are indicated at right. The difference between Δ_EC_ mean values is indicated in black. (F) Kinetochores stained with anti-CenpC, anti-Bub1, and anti-Mad2 antibodies in parental and *bub1 1-23* cells treated with 3.3 μM nocodazole. Scale bars, 500 nm. (G) Histograms of the Δ_EC_ distances between CenpC and Mad1-pT716, Mad2, and Zwilch in parental and *bub1 1-23* cells treated as in (F). Mean (dashed line) and SD values are indicated at right. The difference between Δ_EC_ mean values is indicated in black.

We next sought to establish where in the kinetochore the Mad1:Mad2 complex is recruited. While Bub1 is a bona fide kinetochore receptor for human Mad1:Mad2 complexes (Zhang et al., 2017), the RZZ complex is also implicated (Kops et al., 2005) and may even operate as a second receptor (Silió et al., 2015). To read out the position of Bub1, we used an antibody that recognizes the first 130 aa (referred to as Bub1(N); Figure 6B) and found it positioned 58.9 ± 1.1 nm (n = 2,232) from CenpC and therefore 26.9 ± 1.4 nm to the inside of the Ndc80 head domain (Figure 5C; Table S1). Using antibodies that recognize the carboxy-terminus of Rod (recombinant fragment aa 2,100–2,209, referred to as Rod(C)) and Zwilch (Figures 6A and 6B), we found that while Rod(C) is positioned 14.4 ± 2.1 nm inside Ndc80(N), Zwilch is 10.5 ± 1.3 nm to the outside (Figures 6B and 6C; Table S1). In addition, EGFP-Zw10 was positioned 11.5 ± 3.6 nm outside Rod(C) and 13.4 ± 3.3 nm inside Zwlich (Figures 6A and 6B; Table S1). This is broadly consistent with the arrangement of these subunits within the cryo-EM structure of the RZZ complex (Mosalaganti et al., 2017). These results indicate that most of the RZZ complex is located outside the Ndc80 head domain, and thus is spatially separated from Bub1. We can estimate the distance between Zwilch and Rod by subtracting the CenpC to Rod(C) from CenpC to Zwilch distances, giving 24.9 ± 2.1 nm (Figure 6C; Table S1). This distance is similar to that determined from the cryo-EM structure (Mosalaganti et al., 2017) and indicates high nematic order (N = ∼0.8; Table S2). The high nematic order further justifies the assumption of the molecular ruler to organize components of the outer kinetochore/corona. These data also suggest that within an end-on attached kinetochore, the RZZ complex is not forming a head-to-tail dimer, as suggested by cryo-EM (Mosalaganti et al., 2017); this would have resulted in Rod(C) and Zwilch signals being coincident. These data thus provide the first 3D mapping of the key checkpoint protein platforms (Bub1 and RZZ) relative to the major microtubule attachment factor (NDC80 complex) in the human kinetochore.

### Mad1:Mad2 Complex Can Occupy Two Distinct Positions within the Kinetochore

We next used the RZZ molecular ruler to map the positions of Mad2 and a phospho-epitope in the carboxy-terminus of Mad1(pT716) following treatment with nocodazole. As the corona/outer kinetochore begins expanding into crescent-shaped structures under these (unattached) conditions, we limited our analysis to non-expanded kinetochores (a “proto-corona”) to have accurate positional information (Figure 6D). The position of Bub1, Rod, and Zwilch (RZZ complex) remained largely unchanged (Figure 6C), showing that kinetochores do not simply collapse when unattached. Mad1-pT716 was located 54.4 ± 2.0 nm (n = 911) from CenpC (close to its binding partner Bub1), while Mad2 was located 16.5 ± 2.7 nm further outward (Figures 6C and 6E; Table S1). This distance is consistent the Mad1:Mad2 complex structure (Figure 6E; Allan et al., 2020, Kim et al., 2012, Sironi et al., 2002). Moreover, the distance from Mad1-pT716 to an antibody that recognizes an epitope (aa 77–115) in the amino terminus, referred to as Mad1(N), was 48.4 ± 13.7 nm (n = 235) (Figures 6E and S6A), close to the predicted 57 nm from EM structure (Allan et al., 2020). Thus, Mad1 looks to be extended within the kinetochore and highly ordered (Table S2).

Mad1:Mad2 are proposed to bind to both Bub1 and a second RZZ-dependent receptor, recently identified as CyclinB (Allan et al., 2020, Silió et al., 2015). Our measurement of Mad1:Mad2 would thus reflect the average position of these proteins within a kinetochore. To test this idea, we measured the position of Mad1-pT716 and Mad2 in cells carrying a homozygous *bub1 1-23* mutation (Figures 6F, S7A and S7B). In these cells, Bub1 levels are reduced to almost undetectable levels, but Mad1-pT716, Mad2, and Zwilch can still bind kinetochores and generate a checkpoint signal (Figures S7A, S7B, and S8A) (Currie et al., 2018, Zhang et al., 2019). Here, both Mad1-pT716 and Mad2 signals moved outward by 13.7 ± 2.3 nm and 13.2 ± 2.5 nm, respectively (Figure 6G). In contrast, we found that the Zwilch position from CenpC was 92.1 ± 2.9 (n = 296) in parental cells and 97.2 ± 5.8 (n = 133) in *bub1 1-23* cells, suggesting that the RZZ complex was not affected by the loss of Bub1 (Figure 6G). These data are consistent with the model that there are two spatially distinct kinetochore receptors for Mad1:Mad2.

### Kinetochores Adopt a Unique Conformation following the Loss of Tension

**Figure 7:**
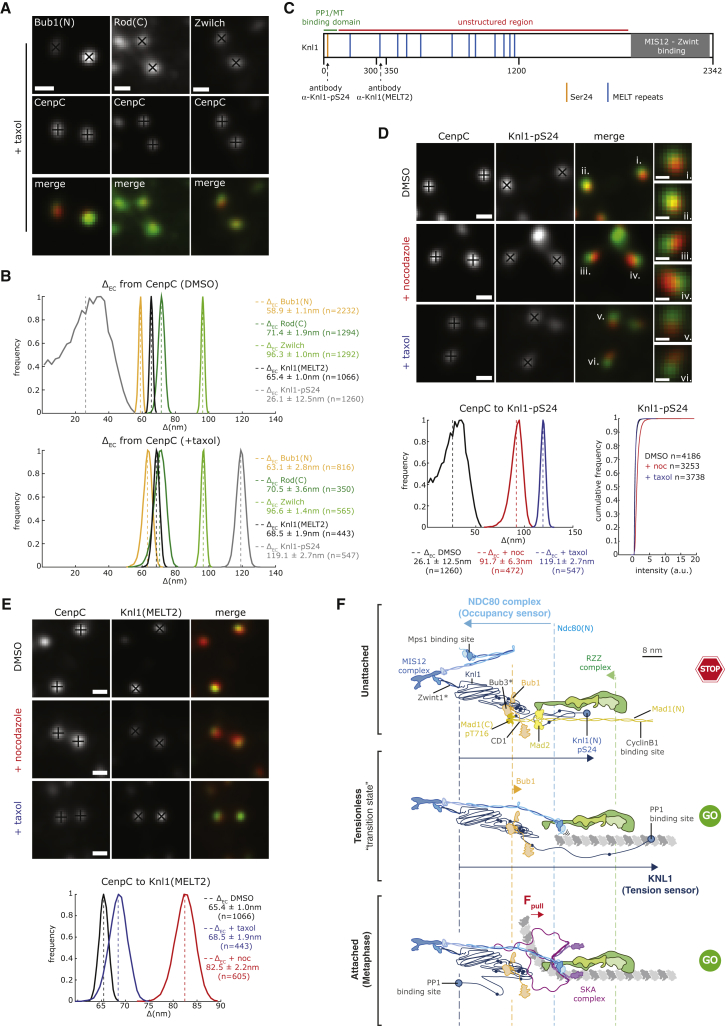
Knl1 1–300 Unravels upon the Loss of Kinetochore Tension (A) Kinetochore pairs stained with anti-Bub1, anti-Rod, and anti-Zwilch antibodies in cells treated with 1 μM taxol for 15 min. Scale bars, 500 nm. (B) Histograms of the Δ_EC_ distances from CenpC to Bub1, Rod, Zwilch, Knl1-MELT2, and pKnl1(S24) in DMSO and taxol. Mean (dashed line) and SD values are indicated at right. (C) Schematic map of Knl1 where the positions of Serine24 (Ser24, orange), MELT repeats (blue), and MIS12-Zwint-binding domain (gray) are shown. Lines indicate the PP1-microtubule binding site (green) and the unstructured region (red). Arrows indicate the binding sites of anti-Knl1-pS24 and anti-Knl1(MELT2) antibodies used in this study. (D) Top: kinetochores stained with anti-CenpC and anti-Knl1-pS24 antibodies in cells treated with 3.3 μM nocodazole for 2 h, 1 μM taxol for 15 min, or DMSO. Scale bar, 500 nm. Insets show enlargements for the indicated kinetochores. Scale bar, 250 nm. Bottom: histograms of the Δ_EC_ between CenpC and Knl1-pS24 in cells treated as above. Mean (dashed line) and SD values are indicated below. Cumulative frequency plots display the Knl1-pS24 intensity in DMSO-, nocodazole (+noc)-, and taxol-treated cells. The Knl1-pS24 signal is normalized to the CenpC signal. Signals are background subtracted. (E) Top: kinetochores stained with anti-CenpC and anti-Knl1(MELT2) antibodies and treated as in (D). Scale bars, 500 nm. Bottom: histograms of the Δ_EC_ distances between CenpC and Knl1 in DMSO-, nocodazole (+noc)-, and taxol-treated cells. Mean (dashed line) and SD values are indicated at right. (F) Schematics representing the ensemble average arrangement of MIS12 complex (dark blue), NDC80 complex (light blue), Bub1 (orange), RZZ complex (green), Mad1:Mad2 (yellow), Knl1 (black), and SKA (purple) in RPE1 cells treated with nocodazole (top), taxol (center), and DMSO (bottom). A single microtubule protofilament is shown in gray and dotted lines represent the position of the indicated complexes in control cells. The arrows indicate the change in position between different conditions. The positions of proteins marked with an asterisk are inferred from known biochemical data. These kinetochore organizational states can be integrated with known checkpoint and error-correction mechanisms as follows: at unattached kinetochores (top), NDC80 is disordered and in an auto-inhibited state, while the first 300 aa of Knl1 are in an extended conformation. Aurora B kinase activity dominates, kinetochore substrates (i.e., Ndc80 and Knl1) are phosphorylated, and Ndc80 is in a low-affinity microtubule-binding state. We note that Mad1 can bridge from the outer kinetochore to the corona, with interactions at either end necessary for stable binding and spindle checkpoint activation. When end-on attachment forms (center), but no tension is generated (e.g., monotelic attachments in which one sister is attached and the other is unattached), the checkpoint is silenced on the attached sister concomitantly with the straightening of Ndc80, which binds to the microtubule lattice. As force from the microtubule depolymerization generates tension (bottom), SKA complexes are recruited and the Knl1 amino terminus ravels. PP1 now fully binds, leading to kinetochore dephosphorylation (counteracting Aurora B) and increased microtubule binding affinity. This model can also explain error correction following the loss of tension at a bi-oriented kinetochore, which would switch the system into the transition (center panel) state (see Discussion for details).

The absence of NDC80 jackknifing following taxol treatment raised the question of whether the loss of tension is detected at all. We first checked the position of Bub1 and RZZ subunits in taxol-treated cells and found no changes (Figures 7A and 7B). We then turned to Knl1, the third component of the KMN network (Cheeseman et al., 2006). Knl1 is a largely disordered protein that binds to the MIS12 complex through the carboxy-terminus with the remaining protein comprising multiple MELT sequences that operate as phospho-dependent binding sites for the Bub3-Bub1 checkpoint complexes (Figure 7C; London et al., 2012, Shepperd et al., 2012, Yamagishi et al., 2012). The extreme amino-terminal end of Knl1 contains a microtubule-binding site and a docking site for protein phosphatase 1 (PP1) (Figure 7C). The amino terminus of Knl1 (marked with a phospho-specific antibody that recognizes serine 24, referred to as Knl1-pS24; Figure 7C) was positioned 32.8 ± 12.5 nm from Bub1 (Figure 7B), whereas the second MELT (aa 300–350, referred to as Knl1(MELT2); Figure 7C) was only 6.5 ± 1.5 nm from Bub1, which is consistent with the role of the MELT motifs in recruiting Bub1 to the kinetochores (Figures 7B and 7E). These data suggest that the unstructured region of Knl1 (Figure 7C), which has a predicted path length of ∼380 nm, must be “wrapped up” and occupy the space between the Ndc80 head domain and the CCAN (CenpC here). We next checked the position of the amino terminus of Knl1 (Knl1-pS24) following taxol treatment (Figure 7D) and found that it moved outward by 93.0 ± 12.8 nm (Figure 7B). There was minimal movement of the second MELT, indicating that the bulk of the MELT array is unchanged (Figures 7B and 7E; Table S1). This is consistent with our observation that the Bub1 position, which is a proxy for the MELTs, does not change. Because the predicted length of the disordered first 300 aa is ∼64 nm and the distance from Knl1(MELT2) to Knl1-pS24 in taxol is 50.6 ± 3.3 nm, this region of Knl1 must switch into an almost straight configuration. Compared to the increase in nocodazole treatment, an increase was not detected in the phosphorylation of Knl1-pS24 (Figure 7D), which is a substrate for AuroraB (Welburn et al., 2010). Because nocodazole also causes a loss of tension, we checked the position of Knl1 (Figures 7D and 7E). Knl1 is again more extended, with the amino-terminal end located 91.7 ± 6.3 nm (n = 472) from CenpC (compared to 26.1 ± 12.5 nm, n = 1,260 in DMSO; Figure 7D; Table S1). We also detected an outward movement (17.1 ± 2.4 nm) of the MELT2 position (Figure 7E). These data provide evidence that the physical re-organization of Knl1 responds to the loss of tension.

## Discussion

This work provides an initial architectural map of the human kinetochore by quantifying the relative position (to accuracy 1–10 nm) and movement of major complexes and subunits (see Figure 7F). Our intra-kinetochore distances are corrected for Euclidean distance inflation and interpreted within the context of a kinetochore ensemble of molecules and available structural data. Using a computational simulation, we generated a 3D visualization of a human kinetochore that produces plate-like structures for the outer (MIS12-NDC80) and inner (CenpC) kinetochores, which is reminiscent of that observed in electron micrographs (Brinkley et al., 1992). These data also demonstrate how the inner and outer plates are shifted relative to each other in the direction perpendicular to the microtubule axis. We propose here that the outer kinetochore is highly ordered with molecules aligned along an outer kinetochore axis, presumably parallel to the K-fiber axis. Within the outer plate, the NDC80 complex and the more distal RZZ complex must have a high nematic order, otherwise the ensemble average distances would not be consistent with distances from structural biology. It thus follows that our methods allow structural insights from cryo-EM and X-ray crystallography to be assessed within an *in vivo* context. They also provide a framework for understanding the higher-order ensemble organization of the kinetochore.

We have discovered that there is substantial re-organization of the kinetochore outer plate when microtubules detach (nocodazole). Our analysis shows that this can be explained through a combination of NDC80 jackknifing and loss of nematic order or through a loss of order alone. Two lines of evidence suggest that jackknifing is involved: (1) *in vitro* experiments have directly shown how the NDC80 complex does jackknife in the absence of microtubules around the loop (Scarborough et al., 2019), and (2) mutations in the Ndc80 loop that reduce the degree of jackknifing interfere with microtubule-kinetochore attachment (Zhang et al., 2012). We therefore favor a model in which the loss of attachment triggers NDC80 jackknifing. However, we note that an additional loss of nematic order within the NDC80 complexes is necessary to explain the measured distance changes. This hints at larger-scale reorganization in the outer kinetochore in the absence of microtubule binding. When kinetochores are attached, but no tension is generated, we find that the first 300 aa of Knl1 are unraveled, while NDC80 remains unchanged. What drives cycles of unraveling-raveling in the first 300 aa of Knl1 remains unknown. We also need to consider that taxol and nocodazole do not represent “physiological” conditions, and it will be important to develop assays to follow Knl1/Ndc80 re-organization during the prometaphase-to-metaphase transition.

The three kinetochore architectures defined in this work (attached, unattached, and tensionless) can be interpreted in terms of our present understanding of spindle checkpoint and error-correction mechanisms (see model in Figure 7F). The jackknifed NDC80 state is tightly correlated with the recruitment of Mad2 to kinetochores and activation of the spindle checkpoint, but it is not a downstream consequence, as the state exists when Mps1 is inhibited (Figure 7F, top). The formation of end-on attachment correlates with the NDC80 complexes straightening out and aligning. During this time, the Mps1 kinase is displaced from kinetochores because of the competition with microtubules for binding to the Ndc80 calponin homology domain (Figure 7F, center; Hiruma et al., 2015, Ji et al., 2015). One idea is that the auto-inhibited Ndc80 conformation gates the recruitment of Mad1:Mad2, perhaps by favoring Mps1 binding. This model is different from that presented in budding yeast, which proposes that attachment separates Mps1 from the Knl1 substrate, thus silencing the checkpoint (Aravamudhan et al., 2015). However, at bi-oriented human kinetochores, the Ndc80 head domain (which binds Mps1) and Knl1 MELTs (marked by the average position of Bub1) are only 26.9 ± 1.4 nm apart (Figure 7F, bottom). Nevertheless, conformational changes in Ndc80 appear to be a conserved feature of the mechanisms that monitor microtubule attachment.

Our data also suggest that the loss of tension (taxol) is not sufficient to activate the spindle checkpoint; the tensionless kinetochores do not jackknife NDC80 and have undetectable levels of Mad2 (Figure 7F, center). The detected mitotic delays during metaphase are likely due to a sub-population of unattached kinetochores (which do jackknife NDC80), as previously suggested (Magidson et al., 2016), although we cannot yet formally rule out weak checkpoint signals (without detectable Mad2 binding). We also propose that taxol-treated kinetochores reflect a “transition state” between the attached (and under tension) and unattached states (see model in Figure 7F for details). While the Aurora B site in the now-unraveled amino terminus of Knl1 is dephosphorylated, fluorescence lifetime imaging microscopy-Förster resonance energy transfer (FLIM/FRET) measurements in taxol-treated cells show that the affinity of NDC80 complexes for microtubules is reduced, an event associated with increased Aurora B recruitment (Yoo et al., 2018). This may be explained by the spatial separation between Knl-pS24 and Ndc80(N) that we observe under that condition. We also need to consider the impact of PP1 recruitment to the amino terminus of Knl1, which competes with microtubules (Bajaj et al., 2018). Understanding how changes in NDC80 microtubule-binding affinity and the spatial balance of phosphatase-kinase activity within the kinetochore are related to the unraveling of Knl1 (and other mechanical transitions) will be an important next step.

In conclusion, our data suggest that kinetochores are able to distinguish between changes in tension and microtubule occupancy using in-built occupancy (Ndc80) and tension (Knl1) sensors.

## STAR★Methods

### Key Resources Table

**Table:** 

REAGENT or RESOURCE	SOURCE	IDENTIFIER
**Antibodies**		

Guinea Pig polyclonal anti-CenpC (1:2000)	MBL	Cat#PD030; RRID:AB_10693556
Mouse monoclonal anti-CenpA (1:300)	Enzo	Cat#ADI-KAM-CC006-E; RRID:AB_2038993
Mouse monoclonal anti-Hec1 (9G3, 1:1000)	Abcam	Cat#ab3613; RRID:AB_303949
Mouse monoclonal anti-Bub1 (1:200)	Abcam	Cat#ab54893; RRID:AB_940664
Mouse monoclonal anti-Rod (1:50)	Abcam	Cat#ab56745; RRID:AB_943932
Mouse monoclonal anti-alpha tubulin (1:1000)	Sigma	Cat#T6074; RRID:AB_477582
Mouse monoclonal anti-Ska3 (1:2000)	Santa Cruz	Cat#sc-390326
Mouse monoclonal anti-Mad1 (F-7) (1:500)	Santa Cruz	Cat#sc-376613; RRID:AB_11151587
Rabbit polyclonal anti-alpha tubulin (1:1000)	Thermo Fisher	Cat# PA5-19489; RRID:AB_10984311
Rabbit polyclonal anti-Zwilch (1:1000)	A kind gift from Andrea Musacchio	N/A
Rabbit polyclonal anti-Nnf1 (1:1000)	McAinsh et al., 2006	N/A
Rabbit polyclonal anti-Knl1 (amino acids 300-350 MELT2, 1:500)	Abcam	Cat#ab70537; RRID:AB_1209410
Rabbit polyclonal anti-Mad2 (1:500)	BioLegend	Cat#Poly19246; RRID:AB_2565454
Rabbit polyclonal anti-Knl1-pS24 (1:2200)	Welburn et al., 2010	N/A
Rabbit polyclonal anti-CenpT (1:1000)	Gascoigne et al., 2011	N/A
Rabbit polyclonal anti-Mad1pT716 (1:1000)	Allan et al., 2020	N/A
Goat anti-guinea pig AlexaFluor 647 (1:500)	Invitrogen	Cat#A21450; RRID:AB_2735091
Goat anti-guinea pig AlexaFluor 488 (1:500)	Invitrogen	Cat#A11073; RRID:AB_2534117
Goat anti-guinea pig AlexaFluor 568 (1:500)	Invitrogen	Cat#A11075; RRID:AB_2534119
Goat anti-mouse AlexaFluor 488 (1:500)	Invitrogen	Cat#A32723; RRID:AB_2633275
Goat anti-mouse AlexaFluor 647 (1:500)	Invitrogen	Cat#A21235; RRID:AB_2535804
Goat anti-mouse AlexaFluor 594 (1:500)	Invitrogen	Cat# A11032; RRID:AB_2534091
Goat anti-rabbit AlexaFluor 488 (1:500)	Invitrogen	Cat#A11008; RRID:AB_143165
Goat anti-rabbit AlexaFluor 594 (1:500)	Invitrogen	Cat#A11037; RRID:AB_2534095

**Chemicals, Peptides, and Recombinant Proteins**		

Geneticin (G418)	GIBCO	Cat#10131027
Fugene 6	Promega	Cat#E2691
Oligofectamine	Invitrogen	Cat#12252-011
Nocodazole	Sigma-Aldrich	Cat#M1404
Taxol	Sigma-Aldrich	Cat#PHL89806
DMSO	Sigma-Aldrich	Cat#D2438
DAPI	Sigma-Aldrich	Cat#D9542
VectaShield	Vector	Cat#H-1000; RRID:AB_2336789
Oregon Green (2.5-5μM)	Promega	Cat#G2802
TMR (2μM)	Promega	Cat#G8252
JF549 (250-400nM)	Promega	Cat#GA1110
JF646 (400-800nM)	Promega	Cat#GA1120
SiR-DNA	Spirochrome	Cat#sc007
Reversine	Sigma-Aldrich	Cat#R3904
(S)-MG132	Cayman	Cat#10012628-5

**Experimental Models: Cell Lines**		

RPE1 HaloTag-CenpA (MC148)	This paper	N/A
RPE1 Ndc80-EGFP (MC191)	This paper	N/A
RPE1 EGFP-Zw10 (MC156)	This paper	N/A
RPE1 (MC133)	ATCC	Cat#CRL-4000; RRID:CVCL_4388
RPE1 *bub1 1-23* (MC170)	Currie et al., 2018	N/A
RPE1 Venus-Mad2 (Mad2L1)	Kind gift from Jonathan Pines	N/A
RPE1 Photoactivatable(PA)-GFP-alpha-tubulin (MC021)	Toso et al., 2009	N/A

**Oligonucleotides**		

Small guide RNA (sgRNA) 5′- caccgATGCATGTCAGAAGATCTCT-3′	This paper	N/A
Small guide RNA (sgRNA) 5′- aaacAGAGATCTTCTGACATGCATc-3′	This paper	N/A
Primer: Fwd_Ndc80EGFP 5′- TAAACTGCAGCCATATGTAGTAAC-3′	This paper	N/A
Primer: Rev_Ndc80EGFP 5′- TTGAAATTAGTAAGAAATGAGAGA-3′	This paper	N/A
Primer: EGFPNtRev 5′- CCGGACACGCTGAACTTG-3′	This paper	N/A
Primer: Rev_Ndc80-EGFP 5′- TTGAAATTAGTAAGAAATGAGAGA-3′	This paper	N/A
CenpT siRNA oligo#1 5′- CAAGAGAGCAGTTGCGGCA-3′	Gascoigne et al., 2011 (obtained from Sigma-Aldrich)	N/A
CenpT siRNA oligo#2 5′- GACGATAGCCAGAGGGCGT-3′	Gascoigne et al., 2011 (obtained from Sigma-Aldrich)	N/A
CenpT siRNA oligo#3 5′- AAGTAGAGCCCTTACACGA-3′	Gascoigne et al., 2011 (obtained from Sigma-Aldrich)	N/A
CenpT siRNA oligo#4 5′- CGGAGAGCCCTGCTTGAAA-3′	Gascoigne et al., 2011 (obtained from Sigma-Aldrich)	N/A
CenpC siRNA oligo 5′- GGATCATCTCAGAATAGAA-3′	Klare et al., 2015 (obtained from Sigma-Aldrich)	N/A
All Star RNAi oligo	QIAGEN	Cat#1027281

**Recombinant DNA**		

pX330-U6-Chimeric_BB-CBh-hSpCas9	Addgene	Cat#42230; RRID:Addgene_42230
Ndc80 Homology directed repair construct (HDR)	This paper	N/A
EGFP-Zw10 plasmid (pMC453)	Famulski and Chan, 2007	N/A
HaloTag-CenpA (pMC442)	This paper	N/A

**Software and Algorithms**		

KiT 2.1.10	This paper; Olziersky et al., 2018	https://github.com/cmcb-warwick
Euclidian correction	This paper, on request	N/A
Huygens 4.1 (Deconvolution software)	SVI	N/A
Volocity 6.0 (UltraView microscope software)	PerkinElemer	N/A
MATLAB (2017a and 2018a)	MathWorks	N/A
softWoRX 6.0 (DeltaVision microscope software)	Applied Precision	N/A
SlideBook 6.0 (Marianas microscope software)	3i	N/A
Fiji	Open source	N/A

### Lead Contact and Materials Availability

Reagents generated in this study will be made available on request to the Lead Contact, Andrew McAinsh (A.D.McAinsh@warwick.ac.uk), but we may require a payment and/or a completed Materials Transfer Agreement if there is potential for commercial application.

### Experimental Model and Subject Details

Immortalized (hTERT) diploid human retinal pigment epithelial (RPE1) cells, RPE1 *bub1 1-23* (MC170; (Currie et al., 2018)), RPE1 Ndc80-EGFP (MC191), RPE1 Venus-Mad2 (Mad2L1 Venus/+; kind gift from Jonathan Pines), RPE1 HaloTag-CenpA (MC148), RPE1 eGFP-Zw10 (MC156) and RPE1 expressing photoactivatable PA-EGFP-alpha-tubulin (MC021, Toso et al., 2009) were grown in DMEM/F-12 medium containing 10% fetal bovine serum (FBS), 2 mM L-glutamine, 100 U/ml penicillin and 100 μg/ml streptomycin. 200 μg/ml and 300 μg/ml Geneticin (G418) were added to the media to maintain MC021 and MC148 cells, respectively. All cell cultures were maintained at 37°C with 5% CO_2_ in a humidified incubator.

### Method Details

#### Construction and verification of cell lines

The RPE1 HaloTag-CenpA (MC148) cell line was generated by transfecting RPE1 cell with the HaloTag-CenpA (pMC442) plasmid. After 24hr cells were plated in selective media containing 300 μg/ml Geneticin (G418). Subsequently, positive clones were visually selected after addition of 2 μM TMR. To generate the RPE1 eGFP-Zw10 (MC156) cell line, RPE1 cells were transfected with EGFP-Zw10 plasmid (pMC453). After 24 hr, EGFP-expressing cells were isolated by FACS sorting and single clones were selected by visual inspection. For CRISPR engineered RPE1 cell lines small guide RNAs (sgRNAs) (5′-caccgATGCATGTCAGAAGATCTCT-3′ and 5′-aaacAGAGATCTTCTGACATGCATc-3′) targeting exon 17 of the NDC80 gene were designed using http://zlab.bio/guide-design-resources to insert 3xFlagtag-EGFP in frame just prior to the stop codon. sgRNAs were annealed and ligated into pX330 which enables their expression in mammalian cells along with a humanized S. pyogenes Cas9 (Ran et al., 2013). A homology Directed Repair (HDR) construct was designed with 800bp homology upstream of the Stop codon and 800 bp downstream of the stop codon. 1 μg of sgRNA construct and 1.5 μg of linearized HDR plasmid were transfected into RPE1 cells using Fugene 6. Positive cells were FACS sorted to isolate the Ndc80-EGFP expressing cells and single clones were identified by visual inspection with confocal microscope, with a further round of clonal selection used to eliminate heterogeneity in the population. PCR analysis of clone MC191 confirmed the presence of a wild-type and EGFP-containing allele (primers: Fwd_Ndc80EGFP, 5′-TAAACTGCAGCCATATGTAGTAAC-3′; Rev_Ndc80EGFP, 5′-TTGAAATTAGTAAGAAATGAGAGA-3′). Individual alleles were then analyzed by cloning PCR products and sequencing the products (primers: EGFPNtRev 5′-CCGGACACGCTGAACTTG-3′ for the EGFP containing allele band and Rev_Ndc80-EGFP 5′-TTGAAATTAGTAAGAAATGAGAGA-3′ for the wild-type allele band). This confirmed that the EGFP was in-frame with the 3′ end of NDC80, although a single amino acid change (Threonine 635 to Alanine, T635A) was identified in the unstructured tail distal to the coiled coils that are required for NDC80 complex tetramerisation. We also note that this variant is found in all primates, except *H. sapiens* and H. Neanderthalensis, and that no difference in mitotic timing or multiple delta measurements (Table S1) were detected when compared to parental RPE1 cells (Figure S2). All cell lines were verified by comparing their mitotic timing with parental controls using long term live cell imaging (see below).

#### Drug treatments and siRNA transfection

For drug treatments, cells were plated on glass coverslips (0.16-0.19 mm) 24 or 48 hr before treatment with 3.3 μM nocodazole (diluted in DMSO) for 2 hr, 1 μM taxol (diluted in DMSO) for 15 min or with 0.1% DMSO for 2 hr as a control. In our experimental set-up, we found that treatment with 3.3 μM nocodazole for 15 min tended to leave microtubules stubs in some kinetochores. Thus, we used a 2 hr nocodazole treatment to ensure depolymerization of all microtubules. We note that a 45 min treatment did also eliminated microtubules and produced similar changes in kinetochore organization. Inhibition of Mps1 was performed using 1 μM reversine in the presence of 3.3 μM nocodazole and 10 μM MG132 for 1 hr. As controls, cells were treated with either DMSO and 10μM MG132 or 3.3 μM nocodazole and 10 μM MG132 for 1hr. To deplete CenpT and CenpC, siRNA oligos targeting their mRNA coding sequences were transfected into RPE1 Ndc80-EGFP (MC191) cells and incubated for 48 hr. For CenpT we used 100 nM of 4 pooled siRNA olgos (25 nM each), whereas for CenpC we transfected 60 nM of a single siRNA oligo. As control, we used 100 nM of the siRNA AllStar oligo. Transfection was performed using oligofectamine following manufacturer’s instructions. All depletions were verified by immunofluorescence microscopy (see below).

#### Immunofluorescence microscopy

Cells were then fixed in 10 mM EGTA, 1 mM MgCl2, 20 mM PIPES pH 6.8, 0.2% Triton X-100, and 4% formaldehyde for 10 min, washed 3 times in PBS before incubation in PBS supplemented with 3% BSA for 30 min to block non-specific antibody binding. Next, cells were incubated with primary antibodies for 1 hr, washed 3 times in PBS and then incubated for 30 min with secondary antibodies and DAPI (1:1000 dilution); all antibodies were diluted in PBS + 3% BSA. Cells were then washed in PBS and mounted in Vectashield. For experiments that include Ska staining (i.e., CenpC/Ndc80(C)/Ska3 staining), cells were pre-extracted prior to fixation for 1 min with 10 mM EGTA, 1 mM MgCl2, 20 mM PIPES pH 6.8, 0.2% Triton X-100. Image stacks were acquired using a confocal spinning-disk microscope (VOX UltraView; PerkinElmer, UK) equipped with a 100X / 1.4 NA oil-immersion objective and a Hamamatsu ORCA-R2 camera, controlled by Volocity 6.0 (PerkinElmer) running on a Windows 7 64-bit (Microsoft, Redmond, WA) PC (IBM, New Castle, NY). Image stacks were acquired over 61 z-slices separated by 0.2 μm (for the samples) or over 121 z-slices separated by 0.1μm (for the chromatic shift slide, see below) using the 488, 561, 640 and 405 nm wavelength lasers. Acquisition settings were set in order that the kinetochore signals were typically larger than 50 units above background.

#### Delta distance and intensity measurements

Spinning disc images were exported from Volocity 6.0 in OME.TIFF format (The Open Microscopy Environment, UK) and deconvolved using Huygens 4.1, using point spread functions (PSFs) calculated from 100 nm TetraSpeck fluorescent microspheres using the Huygens 4.1 PSF distiller. Where required, images in the 640 nm wavelength were deconvolved within Huygens 4.1 using a theoretical PSF. Deconvolved images were exported from Huygens 4.1 in r3d format and read into MATLAB using the loci-tools java library (The Open Microscopy Environment). Kinetochores spots were first detected using the 561 or 640 nm channel and then (where appropriate) signals from secondary and tertiary markers were identified (Smith et al., 2016). Initial alignment of all three channels was carried out using images taken from a reference slide (either RPE1 HaloTag-CenpA labeled with Oregon Green, TMR or JF549 and JF646; or anti-CenpA stained with a mix of alexa488, 568 and 647-labeled secondary antibodies) on the day of experiment acquisition (Dudka et al., 2018, Smith et al., 2016). Final outer kinetochore positions were corrected (for chromatic aberrations) per cell so that cell-average intra-kinetochore distance was equal to zero in each the microscope x-, y- and z-coordinates, as is the average orientation previously demonstrated (Dudka et al., 2018, Smith et al., 2016). Kinetochore tracking, sister pairing, 3D delta and intensity measurements were made using KiT (Kinetochore Tracking) v2.1.10. Delta 3D (Δ3D) distances were corrected using an Euclidean correction algorithm (see Methods S1) that outputs the Euclidian corrected delta parameter (termed ΔEC). For measurement of Bub1, Mad2, Mad1-pT716, Zwilch and Knl1-pS24 signal, intensities were background subtracted and then normalized using the CenpC signal as a reference (also background subtracted). For measurement of endogenous Ndc80-EGFP and Venus-Mad2, and CenpT and CenpC within the RNAi experiments, the background subtracted and non-normalized signal is reported.

#### Assay for spindle assembly checkpoint activity

Cells were cultured in four compartment CELLview dish (627975, Greiner Bio-One Ltd.). Time-lapse imaging was performed on an Olympus DeltaVision microscope (Applied Precision, LLC) equipped with a Photometrics CoolSNAP HQ camera (Roper Scientific) and a stage-top incubator (TokaiHit) to maintain cells at 37°C and 5% CO2. Temperature was further stabilized using a microscope enclosure (Weather station; Precision Control) held at 37°C. Image stacks (7 × 2 μm optical sections) were acquired using the softWoRX 6.0 software every 3 min using a 40x / 1.3 NA oil-immersion objective. To visualize the DNA, 1 hr before imaging cells were incubated with DMEM/F-12 media containing 0.5 μM SiR-DNA (Spirochrome). In each experiment, only fields (1024 × 1024 pixels) containing at least one metaphase cells were imaged using the point visit function in softWoRX 6.0. Imaging started after the addition of DMSO or 1 μM taxol-containing media. Cells were imaged for 15 min to reproduce the same conditions used for the fixed cell experiments. For experiments with RPE1 bub1 1-23 cells imaging was extended to 24 min because the timing to anaphase onset was slightly delayed with respect to the parental cell line. To inactivate the Spindle Assembly Checkpoint, cells were treated with either DMSO or 1 μM taxol-containing media for 15 min and then 1 μM reversine-containing media was added for 120 min (total imaging time was 135 min) to inhibit Mps1. As control, cells were treated with 1 μM taxol and imaged for 135 min. Images were acquired at 32% solid source illumination with Cy5 and neutral density filters, exposure time 0.05 s. Timing of exit from mitosis was scored by eye.

#### Long term live-cell imaging

Parental RPE1 and RPE1 stably expressing Ndc80-EGFP were cultured in glass bottom FluoroDish (FD35-100, World Precision Instrument, Inc.). Time-lapse imaging was performed on Olympus DeltaVision microscopes (Applied Precision, LLC) equipped either with Photometrics CoolSNAP HQ (Roper Scientific) or Photometrics CoolSNAP HQ2 cameras (Roper Scientific) and temperature held at 37°C as described above. Image stacks (7 × 2 μm optical sections) were acquired using the SoftWoRX 6.0 software every 3 min using a 40x/1.3 NA oil-immersion objective. To visualize the DNA, 1 hr before imaging RPE1 cells were incubated with DMEM/F-12 media containing 0.5 μM SiR-DNA (Spirochrome). In each experiment, 30 to 40 fields (1024 × 1024 pixels) were imaged using the point visit function in softWoRX 6.0. Images were acquired for 15 hr at 10% solid source illumination with neutral density and Cy5 filter, exposure time 0.05 s. The timing of nuclear envelope breakdown and anaphase onset were scored by eye.

#### Photoactivation experiments

RPE1 cells stably expressing photoactivatable-(PA)-GFP-alpha-tubulin (Toso et al., 2009) were cultured in Fluorodishes (FD35-100, World Precision Instrument, Inc.) and DNA was visualized by incubation for 30 min with CO2 independent L15 media (Invitrogen, UK) containing 0.5 μM SiR-DNA (Spirochrome, CH). DMSO or 1μM taxol was added 15 min prior to imaging. Photoactivation was carried out using a confocal spinning disk microscope (Marianas SDC, 3i, UK) equipped with a Vector module for photoactivation, a 100x / 1.46 NA immersion oil objective and a Photometrics 95B Prime sCMOS camera controlled by Slidebook 6.0 (3i, UK). Cells were maintained at 37°C using a stage top incubator (both Okolab, Italy). PA-GFP-tubulin was activated in an ROI (100x5 pixels, parallel to the metaphase plate) with 4 × 2 ms pulses of a 405 nm laser. Images were then acquired (excitation 488 nm) every 15 s for 2 min (150 ms exposure, 3 planes 1 μm z-step centered on the photoactivated plane). Images of the DNA staining were acquired (excitation 640nm) at frame 1, 5 and 9. Poleward flux was calculated by measuring the displacement of photoactivated marks over the first 5 frames. To determine the turnover of PA-GFP-alpha-Tubulin the background-subtracted pixel intensity of the photo-activated EGFP-alpha-tubulin over time was measured in ImageJ (averaging the mean intensity of two 7x7 pixels square boxes placed on the photo-activated region). In DMSO, intensity was measured at every time point, whereas, in taxol, intensity was analyzed at time points 0 min, 1 min and 2 min.

### Quantification and Statistical Analysis

For all intrakinetochore Δ analysis, expanded kinetochores and kinetochores where the software failed to correctly identify the spot center (as assessed by visual inspection), were excluded to prevent compromising accuracy. All significance tests were done using z-test, except for the microtubule flux rate measurements where we used a t test. Fisher exact test was used to compare the fraction of cells exiting mitosis in live cell imaging experiments. The calculation of Nematic order is defined in Methods S1. In all figures, fluorophores imaged in different experimental conditions are displayed using the same dynamic range for comparison purposes. Effective cell number is calculated as 1/SI where SI is the Simpson Index Σ_i_ p_i_^2^ where p_i_ is the fraction of KTs in cell i.

### Data and Code Availability

Kinetochore Tracking (KiT) 2.1.10 and the Bayesian Euclidean distance correction algorithm (BEDCA) codes are available on our github site: https://github.com/cmcb-warwick

## Supporting Citations

The following references appear in the Supplemental Information: Alushin et al., 2010, Booth et al., 2011, Churchman et al., 2006, Ciferri et al., 2005, Joglekar et al., 2006, Ledbetter and Porter, 1963, Maiolica et al., 2007, Musacchio and Desai, 2017, Petrovic et al., 2014, Craiu and Rosenthal, 2014, Nixon et al., 2015.

## Supplemental Information

Document S1. Figures S1–S8 and Methods S1

Table S1. Comparison of Δ1D, Δ3D, and ΔEC Measurements, Related to Figure 1

Table S2. Nematic Order of MIS12-NDC80, RZZ, and Mad1:Mad2 Complexes, Related to Figure 4

Document S2. Article plus Supplemental Information
