# Supplementary material for: Ensemble-Level Organization of Human Kinetochores and Evidence for Distinct Tension and Attachment Sensors
Source: Cell Rep. 2020 Apr 28;31(4):107535. doi: 10.1016/j.celrep.2020.107535 (PMC7196887; doi:10.1016/j.celrep.2020.107535)
Supplement: Document S1. Figures S1–S8 and Methods S1 [file mmc1.pdf]

Cell Reports, Volume *31*

Supplemental Information

Ensemble-Level Organization of Human Kinetochores and Evidence for Distinct Tension and Attachment Sensors

Emanuele Roscioli, Tsvetelina E. Germanova, Christopher A. Smith, Peter A. Embacher, Muriel Erent, Amelia I. Thompson, Nigel J. Burroughs, and Andrew D. McAinsh

Supplemental figures

Roscioli E., Germanova T., Smith C., Embacher P., Erent M., Thompson A., Burroughs N. J., and McAinsh A. D.

2020

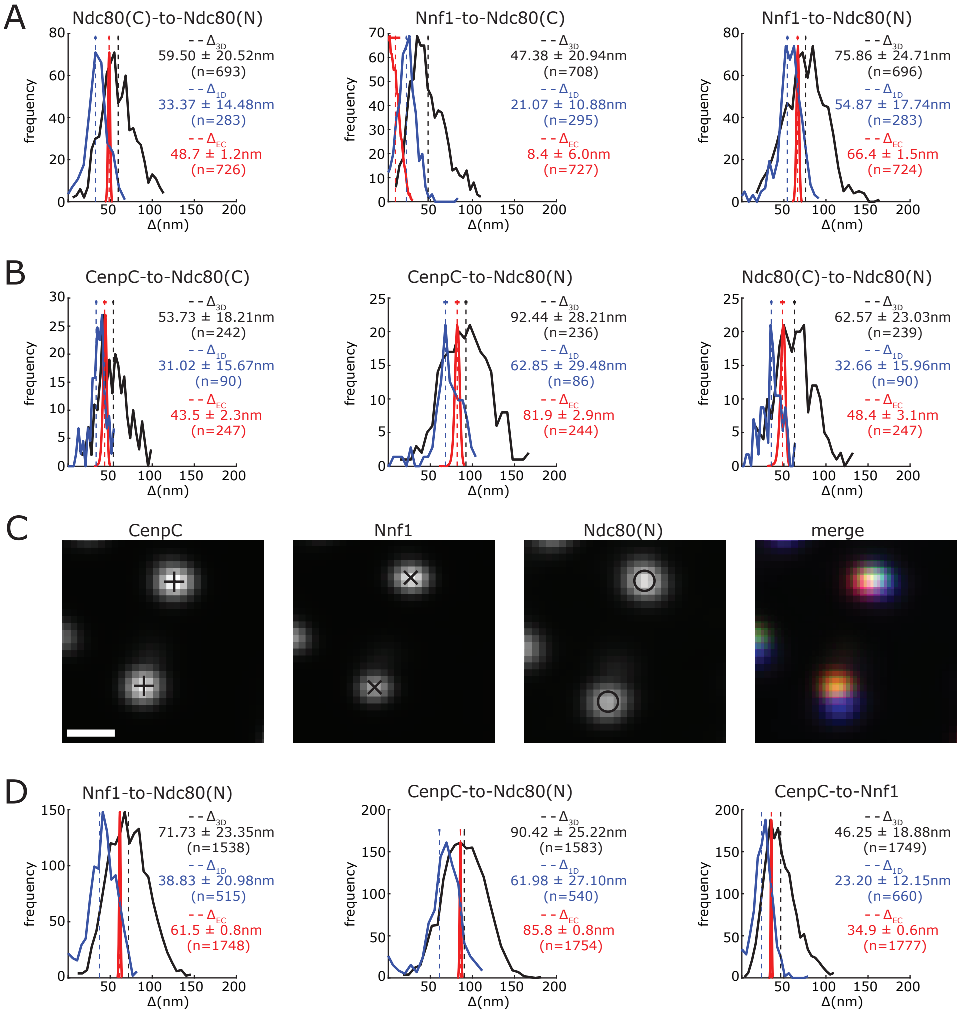

Supplemental Figure 1 related to Figure 1. Comparison of Δ_{1D}, Δ_{3D} and Δ_{EC} measurements. (A) Histograms showing the Ndc80(C)-to-Ndc80(N), Nnf1-to-Ndc80(C) and Nnf1-to-Ndc80(N) Δ_{1D}, Δ_{3D} and Δ_{EC} distances in Ndc80-EGFP cells. (B) Histograms showing the CenpC-to-Ndc80(C), CenpC-to-Ndc80(N) and the Ndc80(C)-to-Ndc80(N) Δ_{1D}, Δ_{3D} and the Δ_{EC} distances in Ndc80-EGFP cells. (C) Kinetochore pair stained with anti-CenpC, anti-Nnf1 and anti-Hec1(9G3) antibodies. Scale bar 500 nm. (D) Histograms show the Nnf1-to-Ndc80(N), CenpC-to-Ndc80(N) and CenpC-to-Nnf1 Δ_{1D}, Δ_{3D} and Δ_{EC} distances in cells. Mean (dashed line) and sd (horizontal bar) values are indicated on the right.

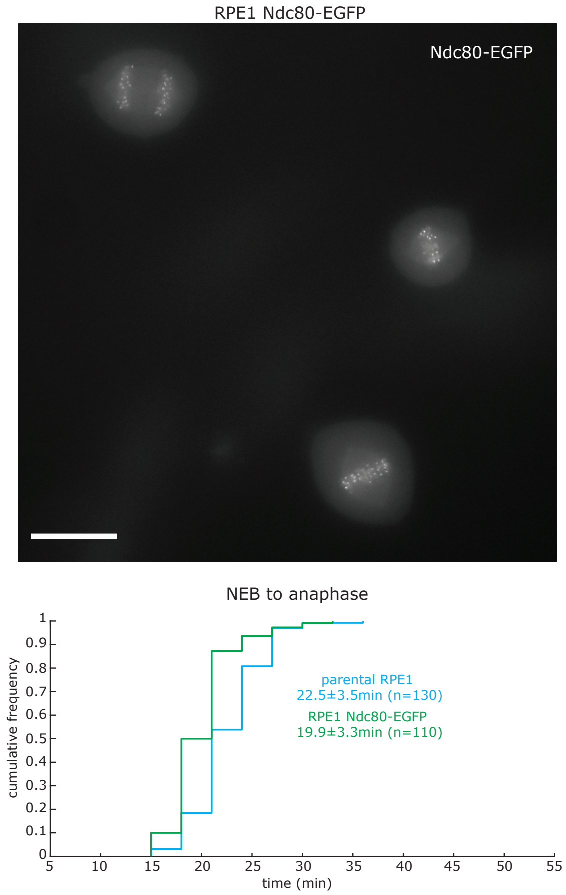

Supplemental Figure 2 related to Figure 1. Parental and Ndc80-EGFP expressing RPE1 cells display the same mitotic timing. Image shows mitotic RPE1 cells expressing Ndc80-EGFP. Scale bar20 μm. Cumulative frequency plot displaying the timing between nuclear envelope breakdown (NEB) and anaphase onset in parental RPE1 and RPE1 Ndc80-EGFP cells (MC191). Mean and sd values are indicated on the right.

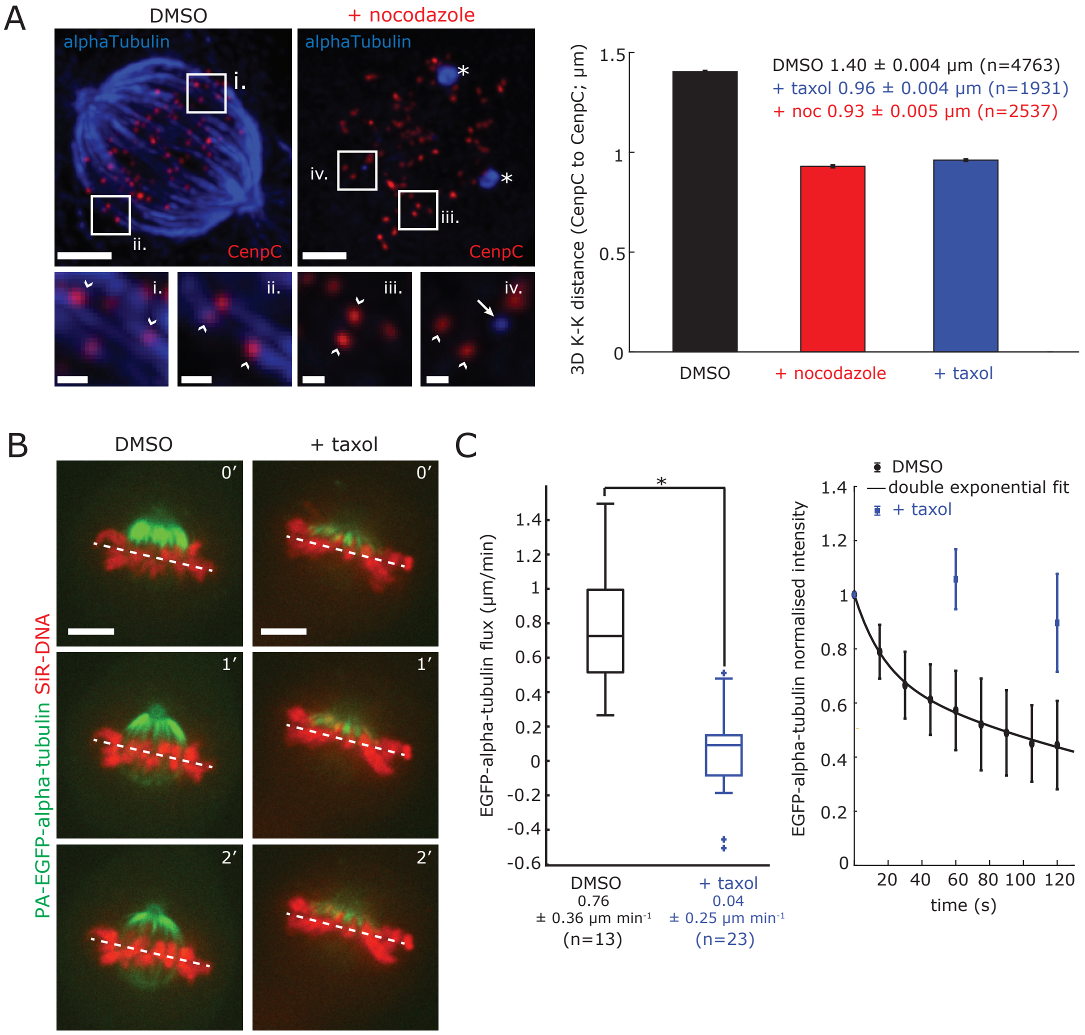

Supplemental Figure 3 related to Figure 4. Nocodazole and taxol reduce inter-sister kinetochore distance and the taxol-dependent loss of inter-sister kinetochore tension is associated with reduced microtubule poleward flux. (A) Example images of RPE1 cells treated with 3.3 μM nocodazole for 2 hr and DMSO as control. Scale bars 3 μm. In control cells, bi-orientated kinetochore pairs (red) are attached to microtubules (blue) and are under tension (white arrowheads in insets i. and ii.; these images were enhanced to better show kinetochores and microtubules). Nocodazole induces full depolymerization of microtubules and reduces the inter-kinetochore distance (white arrowheads in inset iii. and iv.). In this condition, only centrosomes (*) and few rare microtubule stubs (white arrow in inset iv.) remain intact. Scale bar in insets is 500 nm. Bar charts (right) show the 3D K-K distance from CenpC to CenpC in cells treated with 3.3 μM nocodazole for 2 hr, 1 μM taxol for 15 min and DMSO as control. Mean and SEM values are indicated above the chart. (B) Effect of 1 μM taxol treatment on microtubule dynamics was determined by photoactivating PA-EGFP-alpha-tubulin adjacent to the metaphase plate and then measuring its movement to the pole (poleward microtubule flux) and the dissipation of the signal over time (plus-end turnover). Example images of RPE1 expressing photoactivatable PA-EGFP-alpha-tubulin (green) stained with SiR-DNA to visualize the chromosomes (red). Photo-activation was carried out at T=0 and cells were imaged every 15 s for 2 min. White dotted lines indicate the centre of the metaphase plate used as reference to measure the position of the PA-EGFP-alpha-tubulin (green). Scale bar 5 μm. 1 μM taxol abolishes plus-end turnover as the tubulin signal is

stable for 120 s, while the signal in DMSO treated cells exponentially decays as previously reported (Amaro et al., 2010) (C) Left: Box and whiskers plot showing the measured microtubule poleward flux whitin the first 60 s in cells treated with 1 μM taxol or DMSO (see Materials and Methods for details). T-test indicates the difference is significant: (*) p=3.5x10-8. Mean and sd values are indicated below the chart. Right: Intensity of PA-EGFP-alpha-tubulin at the indicated times in 1 μM taxol or DMSO-treated cells (see Materials and Methods for details). Black solid line indicates the double exponential fitting for the DMSO intensities.

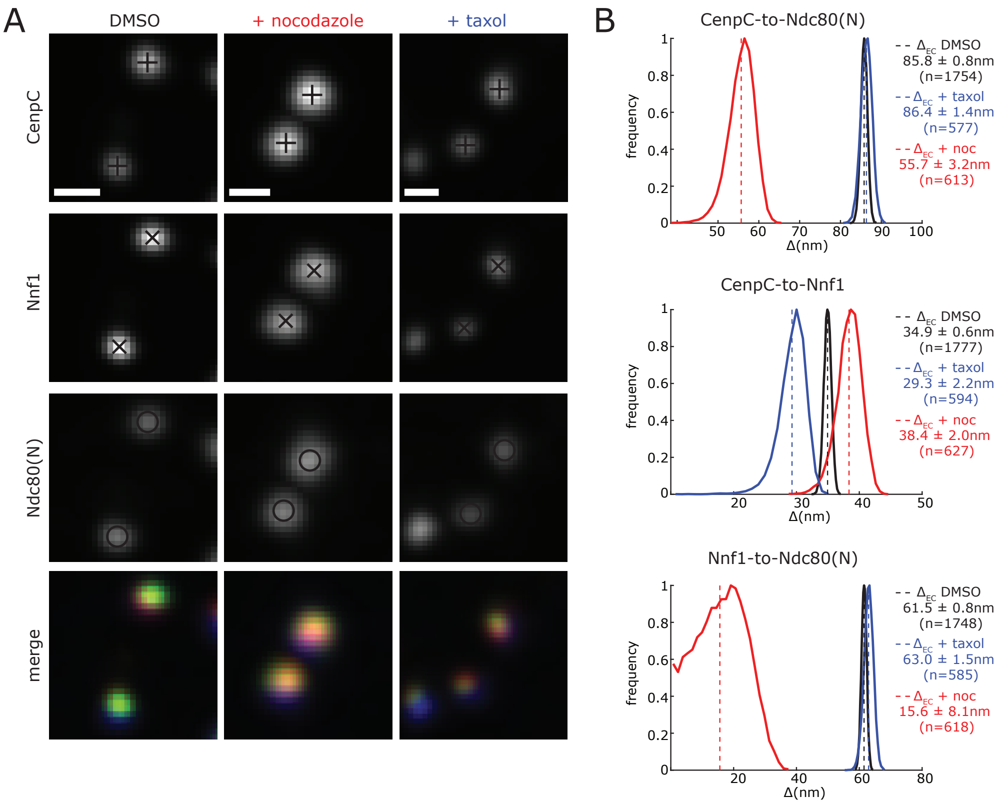

Supplemental Figure 4 related to Figure 4. The Ndc80 N-terminus moves close to Nnf1 in unattached kinetochores. (A) Kinetochores in RPE1 cells stained with anti-CenpC, anti-Nnf1 and anti-Hec1(9G3) antibodies and treated with 3.3 μM nocodazole for 2 hr, 1 μM taxol for 15 min and DMSO as control. Scale bar 500 nm. (B) Distribution of the Nnf1-to-Ndc80(N), CenpC-to-Nnf1 and CenpC-to-Ndc80(N) Δ_{EC} distances in DMSO, nocodazole (+noc) and taxol treated cells. Mean (dashed line) and sd values are indicated on the right.

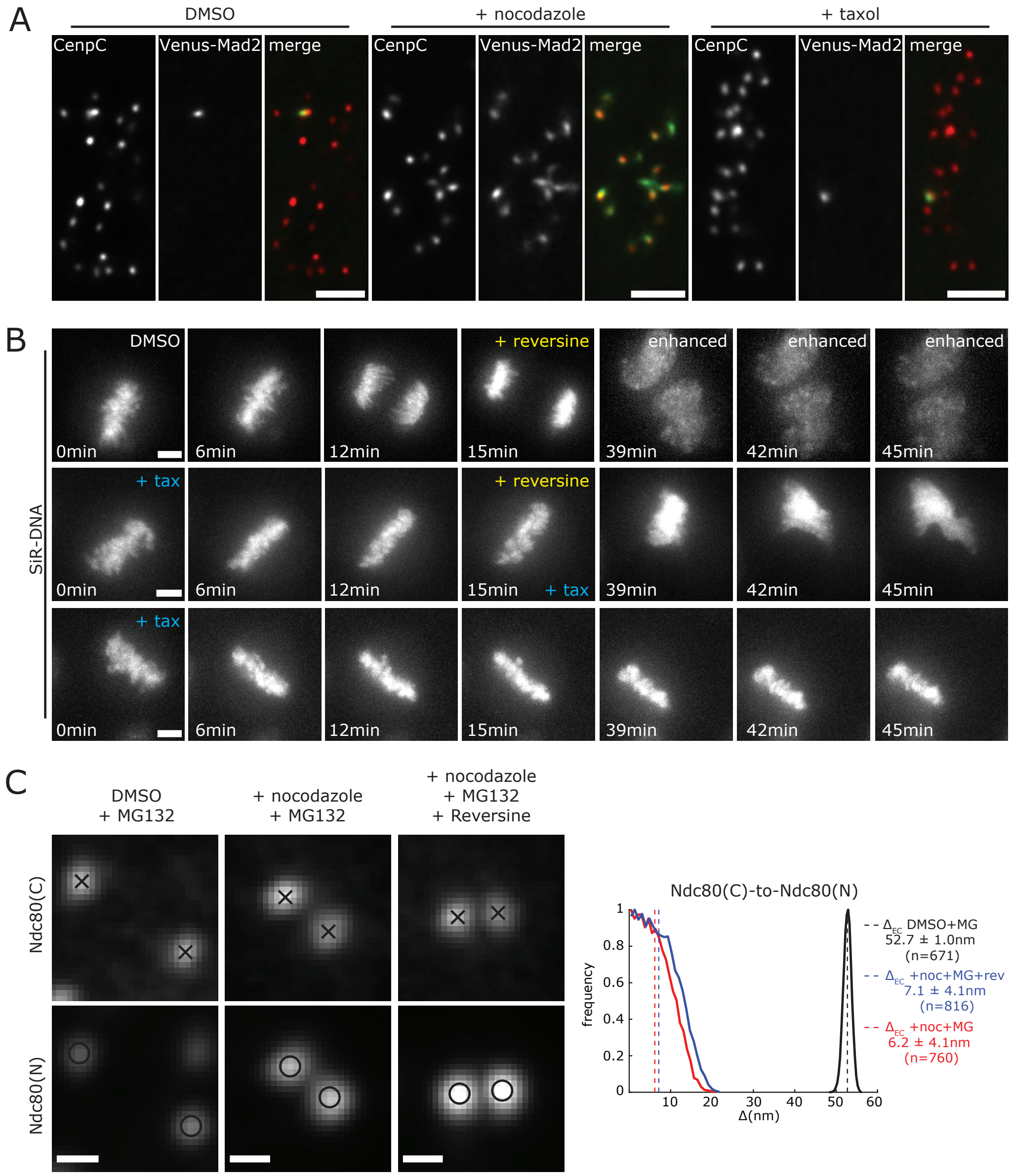

Supplemental Figure 5 related to Figure 5. Taxol-induced mitotic delay is dependent on the activation of the Spindle Assembly Checkpoint. (A) Images of RPE1 Venus-Mad2 cells stained with anti-CenpC antibody and treated with 3.3 μM nocodazole for 2 hr, 1 μM taxol for 15 min and DMSO as control. Scale bars 3µm. (B) Images of RPE1 cells treated with 1 μM taxol (+tax) or DMSO for 15min and then with 1 μM reversine for 2h. Bottom row shows a cell treated with 1 μM taxol (+tax) only, as negative control. Time frame images at 39, 42 and 45 min in the DMSO sample were enhanced to better display the cell exiting mitosis. To visualize DNA, cells were previously treated with SiR-DNA. Scale bars 5 μm. (C) Kinetochore pair in RPE1 Ndc80-EGFP cells stained with anti-Hec1(9G3) antibodies and treated with 3.3 μM nocodazole + 10 μM MG132 for 1 hr, 3.3 μM nocodazole + 10 μM MG132 + 1 μM reversine for 1 hr and DMSO as control. Scale bar 500 nm. Histograms show the distribution of the Ndc80(C)-to-Ndc80(N) Δ_{EC} in cells treated as above. Mean (dashed line) and sd values are indicated on the right.

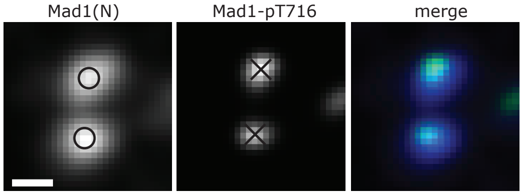

Supplemental Figure 6 related to Figure 6. Mad1(N) to Mad1-pT716 Δ_{EC} distance reflects EM structural data. Kinetochore pair stained with antibodies against Mad1 (aa 77-115), here referred as Mad1(N), and Mad1-pT716. Scale bar 500 nm.

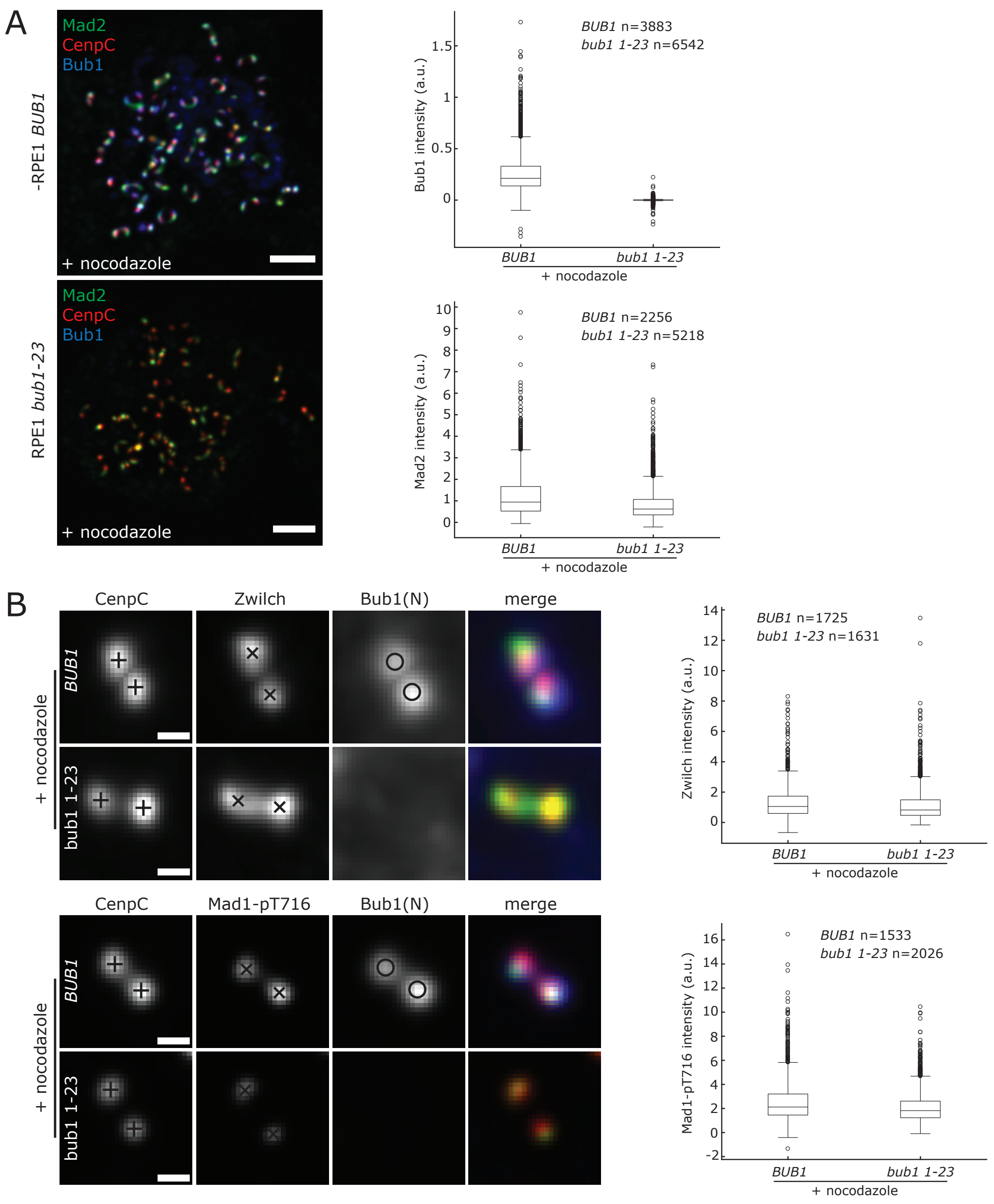

Supplemental Figure 7 related to Figure 6. bub1 1-23 cells can recruit Mad1:Mad2 at kinetochores and are delayed in metaphase upon taxol treatment. (A) Images of parental and RPE1 bub1 1-23 cells stained with anti-CenpC, anti-Bub1 and anti-Mad2 antibodies and treated with 3.3 μM nocodazole for 2 hr. Scale bars 500 nm. Box and whiskers plots on the right show Bub1 and Mad2 intensities in parental and RPE1 bub1 1-23 cells. Bub1 and Mad2 signals were background-subtracted and normalised to CenpC signal (also background corrected). (B) Kinetochore pair stained with anti-CenpC, anti-Zwilch and anti-Bub1(N) antibodies (top) and with anti-CenpC, anti-Mad1-pT716 and anti-Bub1(N) antibodies (bottom) in parental and RPE1 bub1 1-23 cells treated with 3.3 μM nocodazole for 2 hr. Scale bars 500 nm. Box and whiskers plots on the right show Zwilch and Mad1-pT716 intensities in parental and RPE1 bub1 1-23 cells. Zwilch and Mad1-pT716 signals were background-subtracted and normalised to CenpC signal (also background corrected).

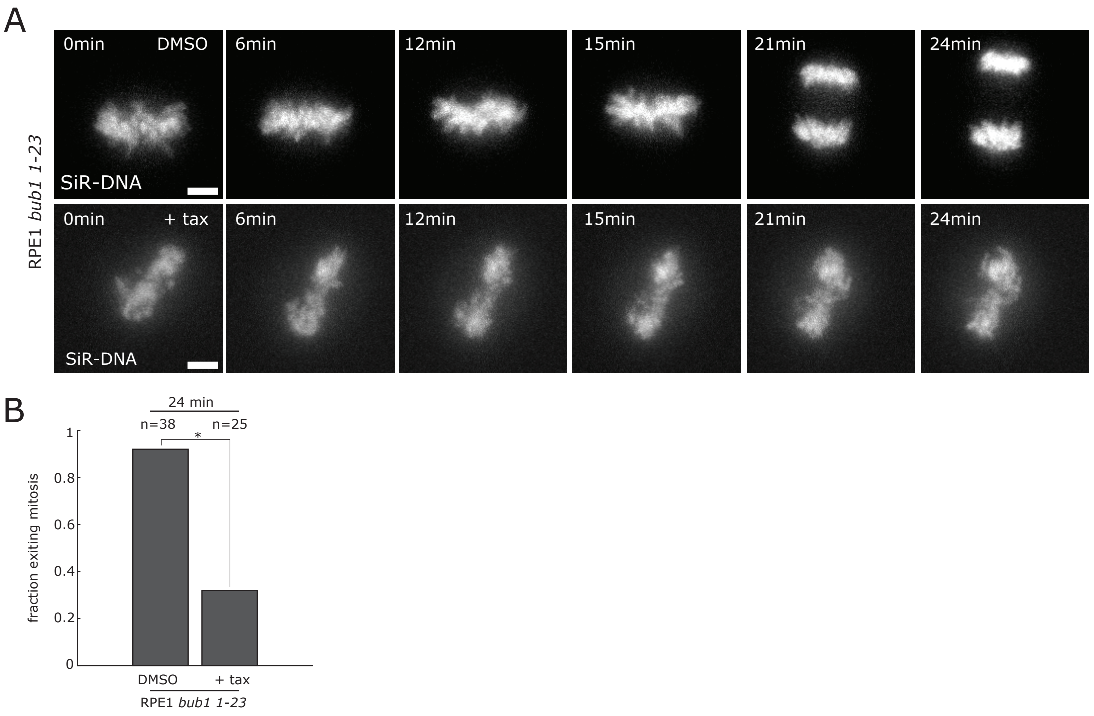

Supplemental Figure 8 related to Figure 6. bub1 1-23 cells activate Spindle Assembly Checkpoint in taxol. Images of RPE1 bub1 1-23 cells treated with 1 μM taxol (+tax) and DMSO as control, for 24 min. To visualize DNA, cells were previously treated with SiR-DNA. Scale bars 3 μm. Bar chart shows the fraction of cells exiting mitosis within 24 min. Fisher's exact test indicates the differences are significant with 99% confidence interval: (*) p=7x10-7.

Methods S1: Δ_{EC} algorithm, Simulation and Nematic order calculations, related to STAR Methods.

Roscioli E., Germanova T., Smith C., Embacher P., Erent M., Thompson A., Burroughs N. J., and McAinsh A. D.

2020

1 A Bayesian Euclidean distance correction algorithm: paired fluorophores

1.1 Introduction

Errors in the measurement of distances causes an over estimation of those distances which is non-negligible when measurement noise is of a similar order to the true distance, see Figure 1. Thus, distances below the resolution of light will be subject to overestimation/inflation under super resolution methods, the degree of inflation being determined by the spatial accuracy of the measurement. Inflation affects measurements in all dimensions, 1D, 2D and 3D, Churchman et al. (2006). Inflation occurs because measurement error can exceed the actual distance, which could then reverse the orientation along a given axis corresponding to a negative displacement (Figure below, black). Since orientation is however not known, eg kinetochores can twist beyond 90°, Smith et al. (2016), the euclidean distance is used which is always positive. This is subject to the same effect, i.e. there is a shift of the measurements upwards because of noise (Figure below, blue).

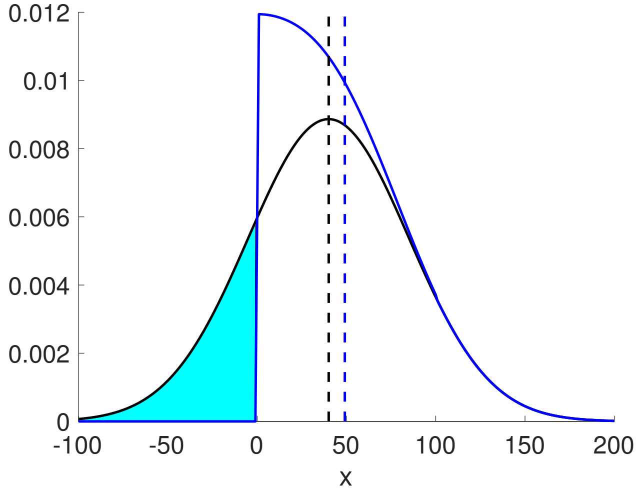

Schematic showing inflation under measurement noise of the displacement between 2 fluorophores in 1D. Histograms of displacement measurements under Gaussian noise assuming known orientation (black), and distance distribution under the modulus (absolute value) operation, $|x|$ when orientation unknown (blue). The measurements where noise exceeds the true distance - switching the orientation of the fluorophores (turquoise shaded) - are measured as positive distances causing inflation, e.g. the mean of the distribution (dashed line) is increased.

From multiple samples of intra-kinetochore distance measurements between two fluorophores we present a Bayesian algorithm to infer the true 3D distance. Noise in the measurement means that the measured distance is an over-estimate, e.g. see (Churchman et al., 2005, 2006), a bias that needs to be corrected. Measurement noise comes from both the microscope point spread function (PSF) and the number of emitted photons, which is determined by the brightness of the flourophores. Thus, measurement noise is both wavelength dependent and protein density dependent. Isotropic measurement errors have been analysed before with published formulae/maximum likelihood correction algorithms in 1-3D (Churchman et al., 2006). However, non-isotropic measurement errors have not been analysed.

In section 1.2 we introduce a 3D model of paired fluorophore measurements. In section 1.3 we derive the associated likelihood. In section 1.4 we present a Markov chain Monte carlo (MCMC) algorithm to sample the posterior distribution of the model parameters. In section 1.5 we demonstrate the algorithm's accuracy on simulated data.

1.2 Euclidean distance model

Let $\vec{X}$ be the observed vector from flourophore 1 to fluorophore 2. For N measurements, we have samples $\vec{X}_i$, $i = 1, 2...N$. Assume that the measurement noise is Gaussian, and if the true displacement is $\mu\vec{n}_i$, with true direction $\vec{n}_i$ (unit vector) and true distance μ, we have

$$\vec{n} \sim \mathcal{P},\ \vec{X}_i \sim \mu\vec{n} + N(0, 2\Sigma^{-1})$$

where Σ is the 3D precision matrix (inverse of covariance) for the spot centre accuracy, and direction vector $\vec{n}_i$ is chosen according to a direction distribution $\mathcal{P}$, e.g. uniform over a sphere. The task is to determine the mean distance $\mu \geq 0$ and the measurement error covariance matrix $2\Sigma^{-1}$. The factor of 2 comes from the fact that both fluorophores have measurement error, so we could write this as the sum of the two measurement errors $2\Sigma^{-1} = \Sigma_1^{-1} + \Sigma_2^{-1}$; however we don't estimate these separately. For isotropic measurements Σ is diagonal; $\Sigma^{-1} = \frac{1}{2}\sigma^2 I$, I is the diagonal matrix and σ is the intradistance error standard deviation. Thus, if spot accuracies are identical, individual spot centres would have variance $\sigma^2/2$. Note that the measurement noise can reverse the orientation, i.e. $\vec{X}_i \cdot \vec{n}_i$ can be negative (see introduction section 1.1).

To quantify distances, it is natural to consider the distribution of $r = |\vec{X}|$; this involves integrating over the orientation distribution $\mathcal{P}$ and the angular components of $\vec{X}$ (i.e. angular measurements are ignored). For the case of isotropic errors we take advantage of the rotational invariance, i.e. choose axes relative to $\vec{n}$; thus, the only coordinate is r - the distance between the fluorophores. Then, for isotropic measurement error in 3D we obtain the probability density (Churchman et al., 2006),

$$\pi_{3D}(r) = \frac{1}{\sqrt{2\pi}} \frac{r}{\mu\sigma} \left(e^{-\frac{(r-\mu)^2}{2\sigma^2}} - e^{-\frac{(r+\mu)^2}{2\sigma^2}} \right) \tag{1}$$

This gives for the mean and variance,

$$E[r] = \sqrt{\frac{2}{\pi}} \sigma e^{-\frac{\mu^2}{2\sigma^2}} + \frac{\sigma^2 + \mu^2}{\mu} erf\left(\frac{\mu}{\sqrt{2}\sigma}\right),\ var(r) = 3\sigma^2 + \mu^2 - E[r]^2,$$

which can, for instance, be fitted to data and μ, σ thereby inferred.

1.3 The model likelihood

For N 3D measurements the likelihood of this model is given by,

$$L = \prod_{i=1}^{N} \left(\frac{1}{(2\pi)^{\frac{3}{2}} \sigma_x^2 \sigma_z} \right) \exp - \left(\vec{X}_i - \mu\vec{n}_i \right)^T \Sigma \left(\vec{X}_i - \mu\vec{n}_i \right),$$

where $2^{-1}\Sigma$ is the 3D precision matrix for the spot centre accuracy (averaged over the two flourophores), and in microscope coordinates is given by $\Sigma^{-1} = diag\left(\frac{1}{2}\sigma_x^2, \frac{1}{2}\sigma_x^2, \frac{1}{2}\sigma_z^2\right)$.

We use spherical coordinates to specify the relative position of the two vectors $\vec{X}_i, \vec{n}_i$; ϕ_i is the relative angle in the x, y plane between $\vec{X}_i, \vec{r}_i$, and θ_i, θ_{Xi} are the angles of $\vec{n}_i, \vec{X}_i$ with the z-axis:

$$\vec{n}_i = (\sin\theta_i, 0, \cos\theta_i),\ \ \vec{X}_i = r_i(\sin\theta_{Xi}\cos\phi_i, \sin\theta_{Xi}\sin\phi_i, \cos\theta_{Xi})$$

Then the likelihood reads,

$$L = \prod_{i=1}^{N}\left(\frac{1}{(2\pi)^{\frac{3}{2}}\sigma_x^2\sigma_z}\right)\exp -\frac{1}{2\sigma_x^2}\left(\mu^2\sin^2\theta_i + r_i^2\sin^2\theta_{Xi} - 2\mu r_i\sin\theta_i\sin\theta_{Xi}\cos\phi_i\right) - \frac{1}{2\sigma_z^2}\left(\mu\cos\theta_i - r_i\cos\theta_{Xi}\right)^2. \tag{2}$$

This has global parameters μ, σ_x, σ_z and kinetochore specific hidden (unmeasured) variables θ_i, ϕ_i, $i = 1, 2...N$ to determine (recall r_i, θ_{Xi} are measured).

1.4 Markov chain Monte carlo methods for Bayesian parameter inference

The objective of Bayesian inference is to determine the probability distribution of the model parameters given the data, specifically the posterior density:

$$\pi(\mu, \sigma_x, \sigma_z, \{\vec{n}_i\}|\{\vec{X}_i\}) = \pi(\{\vec{X}_i\}|\mu, \sigma_x, \sigma_z, \{\vec{n}_i\})\pi(\mu, \sigma_x, \sigma_z, \{\vec{n}_i\})/\pi(\{\vec{X}_i\})$$

from Bayes theorem, where the first term is the likelihood L above and $\pi(\mu, \sigma_x, \sigma_z, \{\vec{n}_i\})$ are the priors on the parameters. Here we are inferring the model using all the kinetochore data, indicated by the set $\{\vec{X}_i\}$, $i = 1, ...n$, i.e. the model assumes all kinetochores are in the same state. This could correspond to all attached, or all detached for example. Using the spherically symmetric measure (solid angle) $d\Omega = \sin\theta d\theta d\phi$, the posterior distribution is thus given up to proportionality,

$$\begin{aligned}\pi(\mu, \sigma_x, \sigma_z, \vec{n}_i|\vec{X}) \quad &\propto \quad \pi(\mu)\pi(\sigma_x, \sigma_z)\prod_{i=1}^{N}\pi(\theta_i, \phi_i)r_i^2\sin\theta_i\left(\frac{1}{(2\pi)^{\frac{3}{2}}\sigma_x^2\sigma_z}\right)\\ &\exp -\frac{1}{2\sigma_x^2}\left(\mu^2\sin^2\theta_i + r_i^2\sin^2\theta_{Xi} - 2\mu r_i\sin\theta_i\sin\theta_{Xi}\cos\phi_i\right) - \frac{1}{2\sigma_z^2}\left(\mu\cos\theta_i - r_i\cos\theta_{Xi}\right)^2,\end{aligned} \tag{3}$$

where $\pi(\mu), \pi(\sigma_x, \sigma_z)$ and $\pi(\theta_i, \phi_i)$ are appropriate priors. If $\mathcal{P}(\vec{n}_i)$ is uniform over the surface of the sphere, the prior on $\cos\theta_i$ is $U([1, -1])$. This distribution has 3 global variables $\mu, \sigma_{x,z}$, and $2N$ hidden variables ϕ_i, θ_i.

However, the posterior can be marginalised in ϕ_i (integrating out ϕ_i using a uniform prior) to give the alternative form with only N hidden variables θ_i,

$$\begin{aligned}\pi(\mu, \sigma_x, \sigma_z, \theta|\vec{X}) \quad &\propto \quad \pi(\mu)\pi(\sigma_x, \sigma_z)\prod_{i=1}^{N}\pi(\theta_i)\sin\theta_i\left(\frac{1}{(2\pi)^{\frac{1}{2}}\sigma_x^2\sigma_z}\right)I_0\left(\frac{\mu r_i\sin\theta_i\sin\theta_{Xi}}{\sigma_x^2}\right)\\ &\exp -\frac{1}{2\sigma_x^2}\left(\mu^2\sin^2\theta_i + r_i^2\sin^2\theta_{Xi}\right) - \frac{1}{2\sigma_z^2}\left(\mu\cos\theta_i - r_i\cos\theta_{Xi}\right)^2,\end{aligned} \tag{4}$$

where I_0 is the modified Bessel function. If the measurement error is isotropic we can also integrate out the angular variables θ_i to obtain the posterior corresponding to (1).

Neither of these posterior distributions are tractable analytically as far as we are aware, and thus numerical methods are required to generate samples. We use a Markov chain Monte Carlo (MCMC) methodology to sample from these posteriors. The marginalised posterior (4) is computationally more expensive per step but has superior mixing than (3) so convergence is achieved with fewer steps. We thus focus on (4) in the following.

There are a vast number of MCMC algorithms, Craiu and Rosenthal (2014). The fundamental idea of MCMC is that parameters are updated either individually, or in groups, using a proposal and these proposals are accepted or rejected such that the distribution is corrected to conform to the conditional posterior. Specifically, consider sampling from the distribution $\pi(\theta)$ (which could be

our posterior above where θ are our model parameters). If the current state of the markov chain is θ, then propose a new $\theta' \sim q(\theta'|\theta)$, where typically a subset of the θ are changed. Then accept the proposal with probability (the Metropolis-Hastings ratio),

$$\alpha(\theta \to \theta') = min\left(1, \frac{\pi(\theta')}{\pi(\theta)}\frac{q(\theta|\theta')}{q(\theta'|\theta)}\right)$$

and remain at θ if the proposal is rejected. This is called the update. A number of proposals are needed so that all parameters are updated. Then under fairly weak conditions the resulting Markov chain converges in probability to the target distribution $\pi(\theta)$, Craiu and Rosenthal (2014). Key to the Metropolis-Hastings ratio is that $\pi(\theta)$ only needs to be known up to normalisation. Special cases include the Gibbs update.

1.5 MCMC sampling algorithm

We use a Metropolis-Hastings within Gibbs algorithm, i.e. we have a mix of Metropolis-Hastings and Gibbs proposals. We use an algorithm that updates variables separately except for μ, σ_x that are highly correlated. We switch to precisions $\tau_{x/z} = \sigma_{x/z}^{-1}$ as is typical for models with Gaussian noise since precisions are (conditionally) Gamma distributed under the choice of a conjugate Gamma prior. We use short hand $s_i = \sin\theta_i$, $c_i = \cos\theta_i$, $s_{Xi} = \sin\theta_{Xi}$, $s_{Xi} = \sin\theta_{Xi}$. We use weak conjugate priors, imposing any positivity conditions by truncation. Updates are as follows:

Joint μ, τ_x proposal. We find that μ and τ_x are often highly correlated. Here we describe a twisted random walk along the eigendirections of the covariance matrix. For an estimated covariance matrix C, determined during burnin (computed sequentially 5 times), define the orthonormal eigenvectors η_j, $C\eta_j = \lambda_j\eta_j$. Then propose a move in the two directions separately, $j = 1, 2$,

$$\begin{pmatrix}\mu \\ \tau_x\end{pmatrix}' = \begin{pmatrix}\mu \\ \tau_x\end{pmatrix} + N(0, \lambda_j)\eta_j$$

which has an acceptance probability,

$$\begin{aligned}\alpha\left(\begin{pmatrix}\mu \\ \tau_x\end{pmatrix} \to \begin{pmatrix}\mu \\ \tau_x\end{pmatrix}'\right) &= max\Big(1, \frac{\pi(\mu')\pi(\tau_x')}{\pi(\mu)\pi(\tau_x)}\left(\frac{\tau_x'}{\tau_x}\right)^N \prod_i \frac{I_0\left(\mu'\tau_x' r_i s_i s_{Xi}\right)}{I_0\left(\mu\tau_x r_i s_i s_{Xi}\right)} \\ &\quad \exp -\frac{1}{2}\tau_x' \sum_i \left((\mu' s_i)^2 + (r_i s_{Xi})^2\right) + \frac{1}{2}\tau_x \sum_i \left((\mu s_i)^2 + (r_i s_{Xi})^2\right) \\ &\quad \exp -\frac{1}{2}\tau_z\left(\mu' - \mu\right)\sum_i c_i\left((\mu' + \mu)c_i - 2c_{Xi}r_i\right)\Big). \end{aligned} \tag{5}$$

The priors enforce rejection of proposals that violate the positivity requirements, $\mu > 0, \tau_x > 0$.

Precision $\tau_z = \sigma_z^{-2}$. Using a conjugate Gamma prior $\Gamma(a_z, k_z)$ we have the Gamma distributed update

$$\tau_z|. \sim \Gamma\left(a_z + \frac{N}{2} - \frac{3}{2}, k_z + \frac{1}{2}\sum_i \left(\mu c_i - r_i c_{Xi}\right)^2\right). \tag{6}$$

Hidden variable θ_i. We use a random walk proposal. We used proposal $\theta_i' \sim \theta_i + N(0, w^2)$, with step size $w = 0.75$ giving reasonable acceptance rates. The acceptance probability is,

$$\alpha(\theta_i \to \theta_i') = min\left(1, \frac{\pi(\theta')\sin\theta_i'}{\pi(\theta)\sin\theta_i}\frac{I_0\left(\mu\tau_x r_i s_i' s_{Xi}\right)}{I_0\left(\mu\tau_x r_i s_i s_{Xi}\right)}\exp -\frac{\tau_x\mu^2}{2}\left((s_i')^2 - (s_i)^2\right) - \frac{\tau_z}{2}\left((\mu c_i' - r_i c_{Xi})^2 - (\mu c_i - r_i c_{Xi})^2\right)\right)$$

Since this is a symmetric random walk, the proposal cancels.

Priors. The prior in μ is a truncated Gaussian ($\mu > 0$) with large standard deviation, $\mu \sim N(60, 100^2)$ (all distances in nm). Priors for τ_x, τ_z are weakly informative Gamma distributions, $\tau_{x/z} \sim \Gamma(a, a\, s^2_{x/z})$ (shape and rate), with $a = 10$. This has mean precision s^{-2} and a relative standard deviation $a^{-\frac{1}{2}} = 0.32$. Weaker priors with wider distributions (smaller a, e.g. $a = 1$) worked on some data sets and gave similar results, but $a = 10$ gave good performance on all our data, see convergence discussion below. For τ_x, τ_z we used $s_x = 20, s_z = 40$ nm respectively when both flourophores are antibodies, and $s_x = 25, s_z = 75$ nm if one of them is a fluorescent protein. Our posteriors are always tighter than these priors and typically have posterior means 20-30 nm for σ_x, and 40-55 nm for σ_z.

1.6 MCMC algorithm performance on simulated data

We tested the algorithm on simulated data (see Figure below). Convergence was determined using the Gelman-Rubin diagnostic for multiple chains and determined converged if the corrected Gelman-Rubin statistic was below 1.1. We used 4 chains with chains initialized from the priors. The true parameters are accurately reconstructed, as seen in the following Figure.

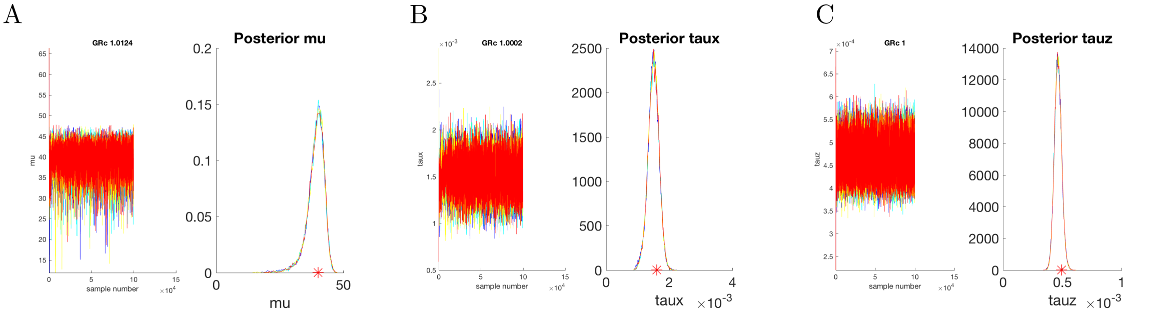

Performance on simulated data. Markov chains (4) and posterior distributions shown for distance μ, precision $\tau_x = \sigma_x^{-2}$, precision $\tau_z = \sigma_z^{-2}$. The four chains are shown in different colors. Corrected Gelman Rubin statistic (GRc) for each variable are indicated on top of panel. True values are indicated by red asterisk on the x-axis, *i.e.* $\mu = 40$ nm, $\sigma_x = 25, \sigma_z = 45$ nm. MCMC runs of length 100000 including 50000 burnin. Priors for $\tau_{x,z}$ had $a = 3$. Data set consists of 1000 simulated kinetochores.

1.7 Running the MCMC algorithm on experimental data

We initially filtered the data with the constraint $|X| < \Delta_{max}$ since extremely large measured distances are clearly in error. For most data we used a threshold $\Delta_{max} = 200$ nm; however for KNL1-pS24 the measured 3D distances still had a significant tail population at 200nm. Thus, we used $\Delta_{max} = 250$ nm for pKNL1 data sets. We reduced the threshold to 180 nm for the Ndc80(C) to Ska distance (7 kinetochores were rejected out of 273) because these outliers caused the precision to be abnormally high.

On all runs we used priors with $a = 10$. We used the Gelman-Rubin diagnostic for 4 multiple chains to assess convergence, requiring that the corrected Gelman-Rubin statistic was below 1.1 on all three parameters μ, τ_x, τ_z (typically below 1.02). Burn-in was 50% of the run in all cases. Most data sets converged within 100,000 steps; for datasets that failed to converge within 100,000 the run length was increased until convergenced attained. The maximum run length (including burn-in) was 2,000,000 for the CenpC-to-Ndc80(C) distance in MC191 cell line under nocodazole treatment. All histograms are based on a single run with 50,000 samples (using subsampling at appropriate rates).

2 Kinetochore architecture simulations

2.1 Structural data

We used kinetochore architectural information and structural data (crystalographic and EM), see Table below, to build a kinetochore simulation. The orientation of the Ndc80/Nuf2 calponin homogy (CH) domains with respect to the microtubule lattice, and the extending coiled coils were from Wilson-Kubalek et al. (2008). The Ndc80 hinge (also called loop or kink) was positioned 16 nm from the CH domains and allowed to bend and rotate given the intrinsic flexibility of the hinge and the coiled coil that connects to the Spc24/Spc25 subunits (Maiolica et al., 2007; Huis in 't Veld et al., 2016; Scarborough et al., 2019; Wang et al., 2008). The Ndc80 hinge angle and the elevation angle of the NDC80 complex short arm (the coiled coil between Ndc80 hinge and Ndc80/Nuf2 CH domains) with respect to the microtubule lattice have been measured using purified complexes but not within the context of the intact kinetochore *in vivo*. So this information was not imposed.

Parameter	Value	References for structural biology
**Microtubule Bundle (K-fibre)**		
K-fibre cross-section area	0.1 μm^2	(Nixon et al., 2015)
Microtubule radius	13 nm	(Ledbetter and Porter, 1963)
Number of microtubules in a K-fibre	20	(Booth et al., 2011)
**NDC80 complex**		
Number of Ndc80 molecules per microtubule	8	(Joglekar et al., 2006)
NDC80 complex full length (along complex)	60 nm	(Huis in 't Veld et al., 2016; Wang et al., 2008; Wei et al., 2005; Screpanti et al., 2011)
Ndc80(C) to Ndc80(N)*	51 nm	estimated from (Huis in 't Veld et al., 2016; Wang et al., 2008; Wei et al., 2005)
Ndc80(C) to Ndc80 hinge	35 nm	estimated from (Huis in 't Veld et al., 2016)
Ndc80 hinge to Ndc80(N)*	16 nm	(Wang et al., 2008)
Spc24/Spc25 RWD domains	4 nm	(Ciferri et al., 2005)
Ndc80(N)* to microtubule axis	4.25 nm	estimated from (Alushin et al., 2010)
**MIS12 complex**		
MIS12 complex full length	20 nm	(Huis in 't Veld et al., 2016; Petrovic et al., 2010)
Nnf1** to Ndc80(C)	14 nm	estimated from (Huis in 't Veld et al., 2016; Petrovic et al., 2010; Ciferri et al., 2005)
Nnf1** to Ndc80(N)*	64 nm	estimated from (Petrovic et al., 2014; Screpanti et al., 2011; Musacchio and Desai, 2017; Huis in 't Veld et al., 2016)

* Ndc80(N) denotes the anti-Hec1 (9G3) antibody binding site (Ndc80 amino acids 200-215; DeLuca et al., 2006)
** The anti-Nnf1 antibody is assumed to bind in the middle of the MIS12 complex

2.2 Simulation

The simulation proceeds as follows. For parameters that are fixed from structural data, the values are described in Section 2.1. Parameters that are fitted to the Ndc80(N)-Ndc80(C)-CenpC triangle distances are indicated below (indicated as 'fitted' in text, and in red color in schematic) and the fitting is described in Section 2.3. Note that variability in angles is set to 10° in absence of constraining data. See schematics below for graphical representation of the incorporated flexibility and annotations.

1. **Microtubule Bundle (K-fibre)**. The K-fibre is defined as a disc of diameter 360 nm, centred on the x-axis. Next, 20 microtubules (MT), radius 13 nm, are uniformly distributed within the K-fibre cross-section. Here, the microtubules lie parallel to the x-axis. If there are overlapping microtubules, all positions are rejected and the process is repeated.

2. **NDC80 complex.** The true mean of the Ndc80 attachment points is labelled x along the central axis of the K-fibre. Next, 8 NDC80 complexes are placed on each microtubule as follows:
   First, we determine a binding site along the MT (Gaussian, mean x, sd 25 nm) and rotate around the MT axis by angle φ_0 (uniform in [0, 360°]). Next, the Ndc80(N) is positioned at 4.25 nm offset from the microtubule surface, considering the 9G3 antibody binding site. This results in 17.25 nm offset from the MT axis (13 nm MT radius). The NDC80 complex short arm is simulated by elevation at angle φ_1 (Gaussian, fitted mean, sd 10°) relative to the microtubule axis, with the Ndc80 hinge oriented towards the inner kinetochore plate. The NDC80 complex short arm can tilt around the axis parallel to the x-axis going through Ndc80(N), angle φ_2 (Gaussian, mean 0°, sd 10°), i.e. the NDC80 complex short arm and the microtubule axes would no longer lie in the same plane for non-zero tilt. The Ndc80 hinge is positioned at a fixed distance of 16 nm from Ndc80(N). The hinge angle between the NDC80 complex short arm and long arm (NDC80 complex coiled coil region between Spc24/Spc25 Head domains and Ndc80 hinge) is defined as φ_3 (Gaussian, fitted mean, sd 10°). We also incorporate rotation of the long arm around the NDC80 complex short arm axis, angle φ_4 (Gaussian, mean 0°, sd 10°). Ndc80(C) is positioned 35 nm from the Ndc80 hinge along the NDC80 complex long arm. Nnf1 is positioned a further 14 nm from Ndc80(C) along the NDC80 complex long arm axis towards the inner kinetochore. Finally, CenpC is positioned as described bellow.

3. **CenpC-Ndc80(N) axis**. Here, we use a model where the inner kinetochore (detected by the CenpC marker in this study) is off axis relative to the K-fibre. The inner-outer kinetochore axis is defined by elevating a line from the x-axis (pinned at x) at angle φ_5 (Gaussian, fitted mean, sd) and rotating around the K-fibre axis by a random angle φ_6 (uniformly distributed). The focus point y is positioned at a fixed distance (fitted) from x along this inner-outer kinetochore axis. CenpC is placed at a distance u (fitted) along the line joining Nnf1 to the focus point y; the focus will act to concentrate CenpC molecules relative to Nnf1. If y is at infinity there is no focusing, i.e. the spread of CenpC and Nnf1 will be identical.

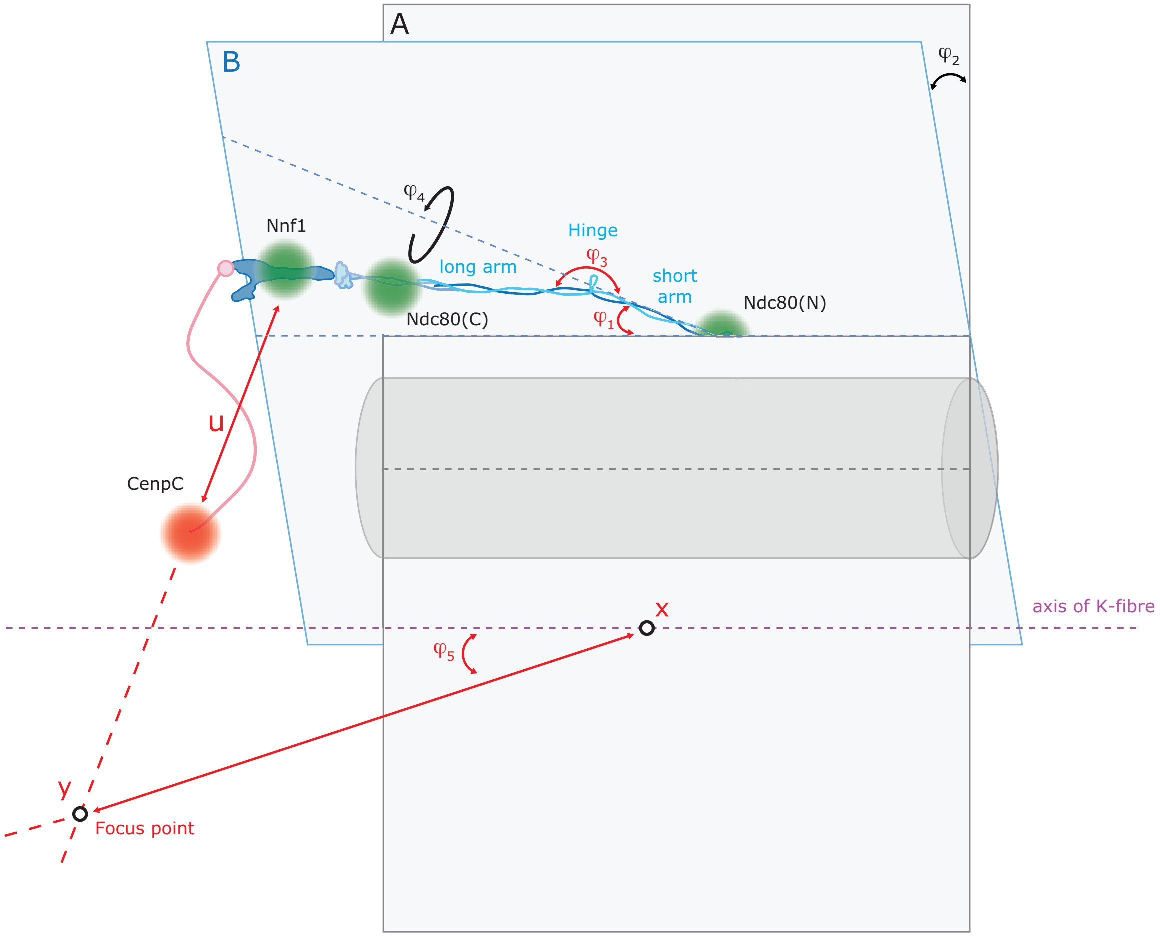

Geometrical setup for simulations of the CenpC-MIS12-NDC80 ensembles. Green and red dots represent the positions of the indicated kinetochore proteins. A representative microtubule is shown in grey and its axis (black dotted line) lies on plane A. The purple dotted line indicates the K-fibre axis (x-axis). The NDC80 complex short arm lies on plane B and tilts (φ_2) around the axis (light blue dotted line) that is parallel to the x-axis and goes through Ndc80(N). Fixed angle parameters used in the simulations are shown in black. Fitted parameters obtained from the simulations are displayed in red. For illustration only, the NDC80 MT attachment is shown vertically above the microtubule ($\varphi_0 = 0$).

2.3 Fitting of parameters. Optimisation.

The measured Δ_{EC} distances in DMSO (cell line MC191) of 1. Ndc80(C)-to-Ndc80(N), 2. CenpC-to-Ndc80(N), and 3. CenpC-to-Ndc80(C), were used to fit six parameters: the focus point y (fitting distance from x, and mean and sd of angle φ_5), the distance u from CenpC-to-Nnf1, the means of the short arm elevation angle and the Ndc80 hinge angle. We use a least-squares fitting method to minimise the difference between simulated distances in the triangle Ndc80(N)-Ndc80(C)-CenpC, and those observed. Specifically we minimise:

$$
\begin{aligned}
V := & \left(\Delta_{sim}\left(\text{Ndc80(N)–Ndc80(C)}\right) - \Delta_{EC}\left(\text{Ndc80(N)–Ndc80(C)}\right)\right)^2 + \\
& \left(\Delta_{sim}\left(\text{Ndc80(C)–CenpC}\right) - \Delta_{EC}\left(\text{Ndc80(C)–CenpC}\right)\right)^2 + \\
& \left(\Delta_{sim}\left(\text{CenpC–Ndc80(N)}\right) - \Delta_{EC}\left(\text{CenpC–Ndc80(N)}\right)\right)^2 ,
\end{aligned}
$$

where the Δ_{sim} are the simulated distances, averaged over 20000 independently simulated kinetochores using the algorithm above.

The optimisation procedure is as follows: Starting from the current values of the 6 parameters a new set of these parameters is proposed based on a random walk (Gaussian) around the current values. The random walk has a drift term $\vec{v}$ so that the algorithm continues to move in profitable directions, where the new drift vector is calculated for each jump and given by,

$$\vec{v}' = \frac{1}{2}\left(\vec{v} + \vec{J}\right)$$

where $\vec{v}$ is the previous drift and $\vec{J}$ is the just accepted jump, if the proposal was accepted, and zero otherwise. The random walk has the following standard deviations: i) 50 nm for the $|x - y|$ distance, ii) 2.5° for all angles except the mean of φ_5, which was 5°. Larger step sizes were used early on to explore a larger part of the parameter space. A proposal is accepted if the cost function V is reduced. Otherwise, the proposal is rejected and a new proposal is attempted. As the simulations have many stochastic degrees of freedom, the Δ_{sim} are prone to fluctuations (despite the 20000 kinetochore sample size). To remove any bias, we re-evaluate V for a new, independent sample of 20000 kinetochores every time after an acceptance and every second time after a rejection. The process is completed if the results are considered close enough to the minimum of V, or if there is no improvement.

This method gives the following fit, reproducing the observed mean distances in the Ndc80(N)-Ndc80(C)-CenpC triangle to within 3 nm:

- angle φ_1: mean 22.4^o. This is within the range 20-60° given by Wilson-Kubalek et al. 2008.
- angle φ_3: mean 203.5^o (where 180° represents straight NDC80 complex conformation and angles $>180^o$ denote clockwise bending).
- distance $|x - y|$: 490 nm
- distance u: 37.9 nm
- angle φ_5: mean 64.2^o, sd 18.1^o.

Here it needs to be noted that if additional constraints are imposed, a solution can still be found that fits the observed distances. For instance, imposing the elevation of the NDC80 complex short arm relative to the microtubule axis (φ_1) to be 10°, sd 10° (restricted to be positive) gives a solution with Ndc80 hinge angle (φ_3) mean of 177.3^o. Therefore, there are nearby solutions that do not require the hinge angle to be above 180°.

3 Nematic order

Nematic order refers to the alignment of the molecules, alignment relative to an orientation axis (the director of Ericksen-Leslie theory). If the alignment distribution is $f(\cos(\theta))$, where θ is the angle between a molecule and the director, alignment order can be quantified in a number of ways. Typically, the distribution $f(\cos(\theta))$ is expanded in Legendre polynomials as follows,

$$f(\cos(\theta)) = \sum_{l=0}^{\infty} \frac{2l+1}{2} N_l P_l(\cos(\theta)).$$

This is analogous to a moment expansion; Legendre polynomials are used because they are orthogonal and $\cos(\theta)$ is valued in $[-1, 1]$. The most common measure of order for uniaxial materials is the second order Legendre polynomial, $N_2 = \mathbf{E}[P_2(\cos(\theta))]$, which can be used for materials with both aligned orientated molecules, eg ferromagnetic, and those with alignment but orientation can be random (no distinguishable head versus tail). For the latter, $f(\cos(\theta))$ is symmetric about zero, so the odd polynomials are zero, including N_1, limiting their general use. The order parameter N_2 is related

to the alignment tensor Q. The first Legendre polynomial order parameter, $N_1 = \mathbf{E}[P_1(\cos(\theta))] = \mathbf{E}[\cos(\theta)]$, can also be used for aligned orientated molecules, with $N_1 = 1$ full alignment, $N_1 = 0$ no alignment. We use this measure because of its easy relationship to the *structural distance fraction*,

$$SDF = \frac{\Delta_{EC}}{\Delta_{structural}}$$

the ratio of the observed (mean) distance between two fluorophores and the expected structural distance $\Delta_{structural}$. Then $N_1 = SDF$. For Ndc80(N)-Ndc80(C) distance the hinge angle needs to be specified. Because each kinetochore is an ensemble, Δ_{EC} is the (average) distance of the average vector over the ensemble. Thus, N_1 is the proportion of the structural distance recovered by the ensemble average. We cannot use our data to compute N_2 since we only have the average ensemble distance Δ_{EC}.

In the simulations nematic order N is defined as ($|\cdot|$ denotes Euclidean distance, $< \cdot >$ denotes average over sample),

$$N = \frac{| < \vec{x} > |}{< |\vec{x}| >}$$

i.e. the length of the average vector to the average length of those vectors. If all molecules (vectors) have the same length then these formulae are identical, i.e. $N = N_1$. They differ when molecule length is not constant, eg for Ndc80 and a variable hinge degree of freedom. If molecule length variation is substantial the SDF and its relation to the order parameter need to be reconsidered as the true structural distance is an ensemble average over these conformations and not known.
